# Spastin depletion increases tubulin polyglutamylation and impairs kinesin-mediated neuronal transport, leading to working and associative memory deficits

**DOI:** 10.1371/journal.pbio.3000820

**Published:** 2020-08-31

**Authors:** André T. Lopes, Torben J. Hausrat, Frank F. Heisler, Kira V. Gromova, Franco L. Lombino, Timo Fischer, Laura Ruschkies, Petra Breiden, Edda Thies, Irm Hermans-Borgmeyer, Michaela Schweizer, Jürgen R. Schwarz, Christian Lohr, Matthias Kneussel

**Affiliations:** 1 Department of Molecular Neurogenetics, Center for Molecular Neurobiology, ZMNH, University Medical Center Hamburg-Eppendorf, Hamburg, Germany; 2 Division of Neurophysiology, University of Hamburg, Hamburg, Germany; 3 Transgenic Animal Unit, Center for Molecular Neurobiology, ZMNH, University Medical Center Hamburg-Eppendorf, Hamburg, Germany; 4 Morphology Unit, Center for Molecular Neurobiology, ZMNH, University Medical Center Hamburg-Eppendorf, Hamburg, Germany; University of Basel, SWITZERLAND

## Abstract

Mutations in the gene encoding the microtubule-severing protein spastin (spastic paraplegia 4 [SPG4]) cause hereditary spastic paraplegia (HSP), associated with neurodegeneration, spasticity, and motor impairment. Complicated forms (complicated HSP [cHSP]) further include cognitive deficits and dementia; however, the etiology and dysfunctional mechanisms of cHSP have remained unknown. Here, we report specific working and associative memory deficits upon spastin depletion in mice. Loss of spastin-mediated severing leads to reduced synapse numbers, accompanied by lower miniature excitatory postsynaptic current (mEPSC) frequencies. At the subcellular level, mutant neurons are characterized by longer microtubules with increased tubulin polyglutamylation levels. Notably, these conditions reduce kinesin-microtubule binding, impair the processivity of kinesin family protein (KIF) 5, and reduce the delivery of presynaptic vesicles and postsynaptic α-amino-3-hydroxy-5-methyl-4-isoxazolepropionic acid (AMPA) receptors. Rescue experiments confirm the specificity of these results by showing that wild-type spastin, but not the severing-deficient and disease-associated K388R mutant, normalizes the effects at the synaptic, microtubule, and transport levels. In addition, short hairpin RNA (shRNA)-mediated reduction of tubulin polyglutamylation on spastin knockout background normalizes KIF5 transport deficits and attenuates the loss of excitatory synapses. Our data provide a mechanism that connects spastin dysfunction with the regulation of kinesin-mediated cargo transport, synapse integrity, and cognition.

## Abstract 2

This study identifies deficits in working and associative memory in spastin knockout mice, resembling the cognitive deficits described in humans with severe forms of SPG4-type hereditary spastic paraplegia. Mechanistically, the findings suggest that impaired microtubule growth, kinesin motility and cargo delivery of synaptic AMPA receptors may contribute to the etiology of the disease.

## Introduction

Hereditary spastic paraplegia (HSP) is a clinically and genetically heterogenous group of neurodegenerative disorders characterized by spasticity and weakness of the lower extremities. With a prevalence of 3–10 individuals per 100,000 inhabitants, HSP is the second-most-important group of motor neuron diseases in most populations [1]. Currently, more than 70 genetic loci of HSP (spastic paraplegia 1 [*SPG1*] to *SPG79*) have been identified, and the pathogenic mechanisms encompass a wide range of cellular dysfunctions, from endoplasmic reticulum (ER) morphogenesis to transport, metabolism, and myelination [1]. Mutations in the spastin gene (*SPG4*) cause the majority of all HSP cases described [2]. Together with mutations in the gene encoding the kinesin family protein (KIF) 5 (SPG10) [1, 3], this suggests a contribution of transport processes in disease etiology.

Mutation analysis of the *SPG4* locus identified various DNA alterations, including nonsense (15%), deletion (23%), insertion (7.5%), splice site (26.5%), or missense mutations (28%) [4, 5], with many of them resulting in loss of function [5]. The *SPG4* phenotype is variable within and among families in terms of severity and age of onset [6]. Originally, SPG4-linked HSP had been considered as a “pure” or “uncomplicated” form, with the main clinical feature of slowly progressive spasticity of the legs.

In addition, later studies described “complicated” forms (complicated HSP [cHSP]), with cognitive impairment, late-onset dementing illness, psychiatric comorbidities, and reduced intelligence quotient (IQ) performance starting at the age of about 40 years [7, 8]. To date, animal models that mimic cHSP with respect to central nervous system (CNS) neurons and brain function have not been described.

The *SPG4* (also known as *SPAST*) gene encodes a protein of the ATPasees associated with various cellular activities (AAA) ATPase family [9]. Spastin is a microtubule-severing protein that breaks longer microtubules into shorter ones. It thereby regulates the number and mobility of microtubules, as well as the total fraction and the distribution of their dynamic plus ends [10, 11]. To extract tubulin heterodimers [12] or to sever microtubules, spastin assembles into hexamers that dock on the filaments and break them by tugging the negatively charged C-terminal tail of tubulin through the central pore of the spastin hexamer [9, 13]. Consistent with this, following knockdown of spastin gene expression in cell culture, the microtubule mass is shifted toward a higher proportion of more-stable regions [14]. A microtubule-severing model therefore proposes that reduced spastin activity causes longer and less dynamic microtubules [9].

Microtubules are hollow tubes typically assembled from 13 laterally associating protofilaments, which in turn consist of α/β tubulin dimers aligned in a head-to-tail fashion. As a consequence of their dimer polarity, they contain plus and minus ends, with plus ends representing the growing ends that associate with microtubule plus-end tracking proteins (+TIPs) (end-binding protein 3 [EB3], among others) [15]. Microtubules are highly dynamic and alternate between rapid phases of growth and shrinkage, a behavior termed “dynamic instability.” They undergo posttranslational modification (PTM), including detyrosination, acetylation, and polyglutamylation, known to alter microtubule function.

Microtubules serve as cytoskeletal tracks for cargo transport through molecular motor proteins, which include plus-end–directed KIFs [16, 17] and minus-end–directed cytoplasmic dynein complexes [18]. Both groups mediate the delivery and/or removal of various cellular cargoes to specific subcellular domains, using ATP as an energy source [19, 20]. Besides other molecular cargoes, KIF5 transports vesicular neurotransmitter receptors, such as α-amino-3-hydroxy-5-methyl-4-isoxazolepropionic acid (AMPA) receptors (AMPARs), along dendrites toward postsynaptic sites [17, 21]. Furthermore, in axons, KIF5 participates in synaptic vesicle transport to presynaptic terminals [22–25].

Because complicated forms of SPG4-linked HSP not only affect motor neurons and motor function but also cause cognitive impairment and dementia [8], we aimed to investigate potential consequences of spastin loss at both central synapses of hippocampal neurons and in learning- and memory-related behavioral tasks. At the molecular and cellular level, it is currently unknown whether the delivery of synaptic vesicles and neurotransmitter receptors to synaptic sites, which requires an intact microtubule cytoskeleton, depends on spastin-mediated microtubule severing. Previous studies using spastin fly and mouse knockout mutants detected axonal transport and outgrowth deficits accompanied by reduced motor control [2, 5, 26, 27] or revealed a role for spastin-mediated microtubule severing in the regulation of microtubule loss during the elimination of neuromuscular junctions [28].

Here, we provide evidence that spastin, in addition to its role in the peripheral nervous system, regulates the function of central neurons in the brain. Our data provide novel mechanistic insights into how spastin loss of function affects polyglutamylation and kinesin processivity, which in turn controls the transport of synaptic cargoes. These parameters are critical in hippocampus-dependent working and associative memory in vivo and may contribute to aspects of cHSP in patients.

## Results

### Spastin knockout causes reduced motor performance

**Fig 1:**
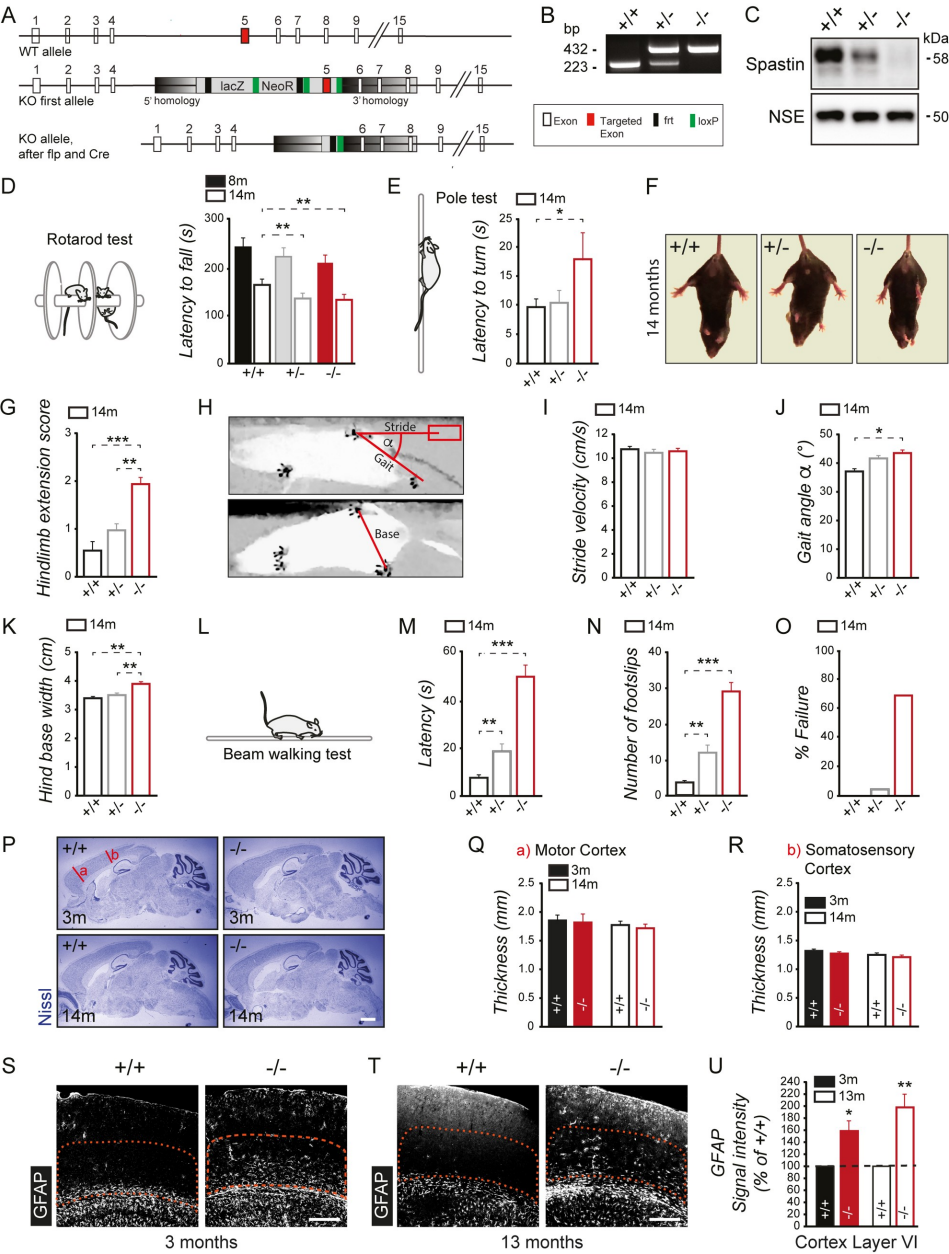
Spastin depletion causes motor impairments in mice. (A) Gene-targeting strategy. Exons of the spastin gene are indicated by white boxes. The targeted exon is marked in red. The gene-targeting cassette (box) with the 5′- and 3′- homology arms (gradient) is shown in gray. The presence of the LacZ reporter and the neomycin resistance (light gray), frt sites (black), and loxP sites (green) before (KO first allele) and after flp and Cre expression (KO allele) are indicated. (B) PCR genotyping. (C) Western blot analysis of spastin levels in adult hippocampal lysates. NSE = loading control. (D) Accelerating rotarod test using 8-month-old (filled bars) or 14-month-old (open bars) mice. Latency to fall off the rod measures balance and motor learning. Eight-month-old mice: main effect for genotype: F_2,61_ = 1.07; *p* = 0.351; followed by pairwise comparisons for (+/+) and (+/−) with *p* = 0.269 and (+/+) and (−/−) with *p* = 0.180; 14-month-old mice: main effect for genotype: F_2,57_ = 3.26; *p* = 0.046; followed by pairwise comparisons for (+/+) and (+/−) with *p* = 0.034 and (+/+) and (−/−) with *p* = 0.035. (E) Pole test using 14-month-old (open bars) mice. Latency to orient downward of the pole measures motor coordination. Main effect for genotype: F_2,57_ = 2.59; *p* = 0.084; followed by pairwise comparisons for (+/+) and (−/−) with *p* = 0.043. (F) Representative pictures of the hind-limb–clasping test using 14-month-old mice. (G) Quantification of the hind-limb–clasping test. Hind-limb extension score measures motor abnormalities. Main effect for genotype: F_2,46_ = 9.48; *p* < 0.001; followed by pairwise comparisons for (+/+) and (−/−) with *p* < 0.001 and (+/−) and (−/−) with *p* = 0.003. (H-K) Gait analysis using 14-month-old mice. (H) Representative bottom view of the gait analysis test. Stride, base, and gait length as well as angle (α) are indicated. Red rectangle indicates the previous position of the hind paw to display the stride length. (I) Quantified stride velocity. (J) Quantified gait angle, main effect for genotype: F_2,46_ = 4.90; *p* = 0.01; followed by pairwise comparisons for (+/+) and (−/−) with *p* = 0.004. (K) Quantified hind base width, main effect for genotype: F_2,46_ = 8.78; *p* = 0.001; followed by pairwise comparisons for (+/+) and (−/−) with *p* < 0.001 and (+/−) and (−/−) with *p* = 0.003. (J-N) (+/+) *n* = 15, (+/−) *n* = 17, (−/−) *n* = 14. (L-O) Beam walking test using 14-month-old mice. (M) Latency to cross the beam indicates motor performance. Fourteen-month-old mice: main effect for genotype: F_2,53_ = 77.839; *p* < 0.0001; followed by pairwise comparisons for (+/+) and (+/−) with *p* = 0.001 and (+/+) and (−/−) with *p* < 0.0001. (N) Number of foot slips while crossing the beam. Fourteen-month-old mice: main effect for genotype: F_2,42_ = 14.368; *p <* 0.006; followed by pairwise comparisons for (+/+) and (+/−) with *p* < 0.006 and (+/+) and (−/−) with *p <* 0.0001. (O) Percentage of failure to cross the beam. Eight-month-old cohort: (+/+) M = 12 mice, (+/+) F = 11 mice; (+/−) M = 10 mice, (+/−) F = 9 mice; (−/−) M = 11 mice, (−/−) F = 11 mice. Fourteen-month-old cohort: (+/+) M = 11 mice, (+/+) F = 12 mice, (+/−) M = 10 mice, (+/−) F = 12 mice, (−/−) M = 9 mice, (−/−) F = 6 mice. (P) Nissl staining of (+/+) and (−/−) sagittal brains sections. Scale bar, 2 mm. Quantified thickness of (Q) motor cortex areas and (R) somatosensory cortex areas derived from 3- and 14-month-old mice as indicated in (P); (+/+) *n* = 11–13, (−/−) *n* = 9–18. (S-U) Immunohistochemistry of cortical sections of (S) 3-month-old and (T) 13-month-old spastin (+/+) and (−/−) mice stained for GFAP to label reactive astrocytes. Note the increase in reactive astrocytes in cortical layer VI (orange region of interest) for spastin (−/−) mice as compared with (+/+) controls; scale bar, 260 μm. (U) Quantification of GFAP signal intensities in cortical layer VI; *n* = 4 mice per group. ANOVA followed by pairwise comparison was used to assess statistical significance. *p < 0.05, **p < 0.01, ***p < 0.001. Student *t* test or ANOVA followed by Tukey’s pairwise comparison was used to assess statistical significance. Data are represented as means ± SEM. Individual quantitative observations that underlie the data presented in this figure are summarized in S1 Data. F, female; flp, recombinase flippase; frt, flippase recognition target; GFAP, glial fibrillary acidic protein; KO, knockout; LacZ, lac operon coding for beta-galactosidase; loxP, locus of X-over P1; M, male; NeoR, neomycin resistance; NSE, neuron specific enolase; WT, wild-type.

To investigate the in vivo relevance of spastin in the brain, we used spastin knockout mice previously generated in our laboratory [28]. Crossbreeding of floxed spastin animals with Cre-Deleter mice [29] caused prenatal and global depletion of spastin gene expression (Fig 1A). Individual genotypes were identified by PCR analysis (Fig 1B), and the loss of spastin protein was confirmed by western blotting from hippocampal lysate (Fig 1C). In contrast, the expression levels of katanin, another microtubule-severing protein, were not increased, thereby excluding major compensatory effects through this functionally related factor (S1A and S1B Fig). Control experiments further revealed equal brain and body weights across sexes and genotypes (S1C–S1F Fig). At the behavioral level, an initial assessment of locomotor activity in a novel open-field arena revealed similar performance in +/+, +/−, and −/− mice (S2A and S2B Fig). Also, anxiety levels analyzed in an elevated plus maze (S2C and S2D Fig) or the light–dark box test (S2E and S2F Fig) turned out to be equal across genotypes.

A previous mutant study analyzing a truncated spastin protein in mice [5] had reported motor deficits. In addition to this model, spastin knockout animals in our study exhibited a range of abnormalities that resemble some clinical features of patients with SPG4-type HSP [6]. With respect to the developmental progression of the disease, we generated two age cohorts (8 months old and 14 months old) consisting of wild-type and homozygous spastin knockout mice. Heterozygous mice (spastin +/−) were also included because they reflect the genetic condition of human polymorphisms. Initially, we performed several tests to examine the effect of spastin depletion on muscle strength, motor coordination, and balance across both ages. Although spastin −/− animals at 8 months old displayed a moderate tendency for impaired performance, they developed most robust and significant deficits in muscle strength and motor coordination at 14 months old (Fig 1D and S1G and S1H Fig). At this age, spastin −/− mice showed increased latencies to orient downward and descend the length of a pole (Fig 1E). We also observed increased hind-limb clasping (Fig 1F and 1G) and altered gait locomotion (Fig 1H–1K and S1I and S1J Fig) in 14-month-old spastin −/− mice as compared with control animals. Furthermore, spastin depletion significantly increased the number of foot slips, failure rates, and latencies to transverse a narrow beam in the walking beam test (Fig 1L–1O).

To assess whether the observed deficits in motor performance correlated with alterations in brain morphology, we next applied Nissl staining. Our histological analysis of sagittal brain sections indicated no major morphological differences across genotypes and age groups (Fig 1P–1R). However, an immunohistochemical analysis of the motor cortex in spastin-depleted mutants revealed a prominent increase in glial fibrillary acidic protein (GFAP) immunoreactivity, indicative of reactive astrocytes, already at 3 months of age (Fig 1S–1U and S1K and S1L Fig). It is therefore likely that neurodegeneration contributes to the motor deficits observed in this study.

Our data are in line with the phenotype of patients with SPG4-type HSP, confirming that spastin is a critical factor in the regulation of mammalian motor coordination.

### Spastin depletion induces working and associative memory deficits

**Fig 2:**
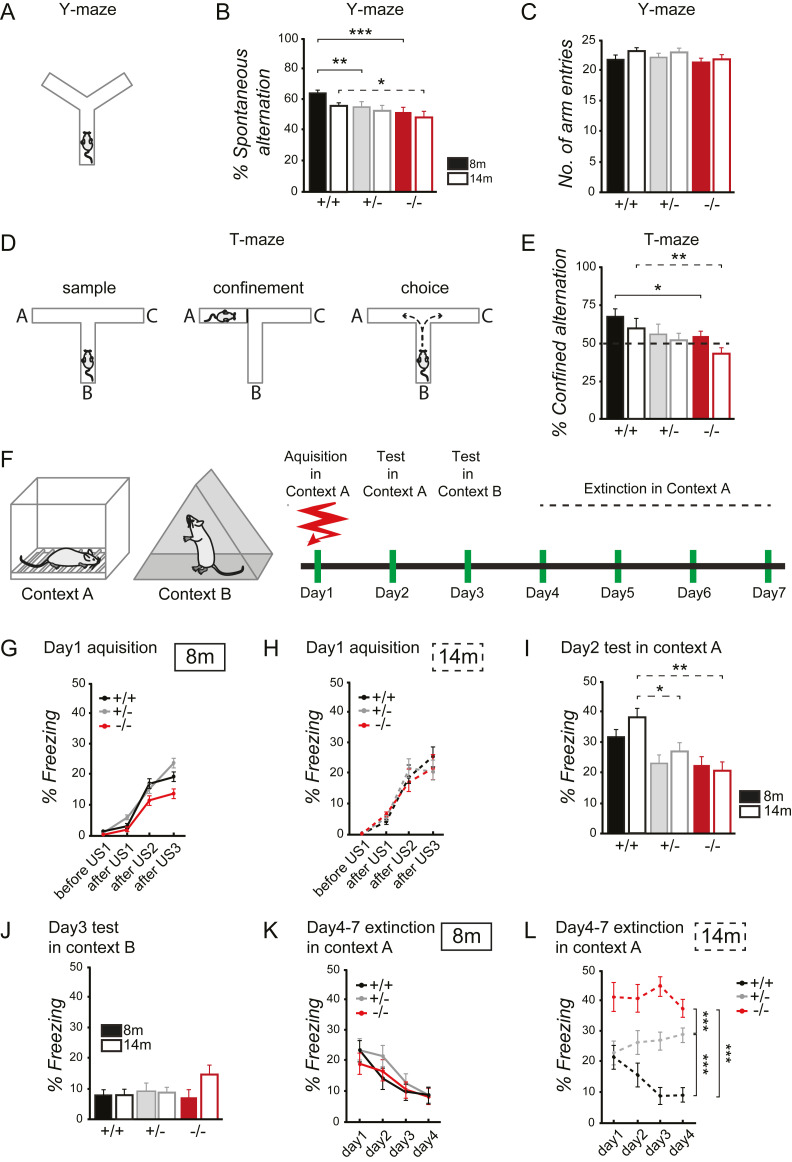
Spastin regulates working memory and associative memory. (A, B) Spontaneous alternation in the Y-maze using 8-month-old (filled bars) or 14-month-old (open bars) mice. Percent alternation between arm entries measures short-term spatial recognition. Eight-month-old mice: F_2,58_ = 12.56; *p <* 0.0001; followed by pairwise comparisons for (+/+) and (+/−) with *p <* 0.001 and (+/+) and (−/−) with *p <* 0.0001; 14-month old mice: main effect for genotype: F_2,55_ = 2.46; *p* = 0.09; followed by pairwise comparisons for (+/+) and (+/−) with *p* = 0.249 and (+/+) and (−/−) with *p* = 0.033. ANOVA was used to assess statistical significance. (C) Explorative behavior, revealed by overall number of arm entries in the Y-maze using 8-month-old (filled) or 14-month-old (striped) mice. Two-way ANOVA (genotype × arm); main effect for genotype: F_2,119_ = 1.649; *p* = 0.197; pairwise comparisons: 8-month-old mice (+/+) and (+/−) with *p* = 0.639 and (+/+) and (−/−) with *p* = 0.615. Fourteen-month-old mice (+/+) and (+/−) with *p* = 0.914 and (+/+) and (−/−) with *p* = 0.090. (D) Experimental design of T-maze experiment. (E) Working and reference memory in the T-maze using 8-month-old (filled bars) or 14-month-old (open bars) mice. Dotted line represents 50% chance level. Confined alternation scores above 50% indicate reference memory of the confined arm. Eight-month-old mice: ANOVA, main effect for genotype: F_1,61_ = 3.04; *p* = 0.055; followed by pairwise comparisons for (+/+) and (+/−) with *p* = 0.066 and (+/+) and (−/−) with *p* = 0.025; 14-month-old mice: ANOVA, main effect for genotype: F_2,46_ = 4.17; *p* = 0.021; followed by pairwise comparisons for (+/+) and (+/−) with *p* = 0.1 and (+/+) and (−/−) with *p* = 0.006. Additional analysis showed that only the (+/+) group alternated at levels significantly above the chance. (+/−) and (−/−) mice performed at 50% chance level or below, respectively; one-sample *t* test against 50% chance level: 8-month-old mice (+/+): *p <* 0.0001; (+/−) *p <* 0.379; (−/−): *p* = 0.321. Fourteen-month-old mice (+/+): *p <* 0.033; (+/−): *p* = 0.727; (−/−): *p* = 0.044. (F) Experimental design of contextual fear-conditioning experiment. (G-H) Percentage time of freezing, indicative of context/shock association, during acquisition at day 1 for 8-month-old mice (G) and 14-month-old mice (H). Two-way ANOVA (genotype × age): F_2,118_ = 0.328; *p* = 0.721; main effect for genotype: F_2,118_ = 1.779; *p* = 0.173. (I) Percentage time of freezing, indicative of conditioned fear memories, at day 2, context A. Eight-month-old mice: ANOVA, main effect for genotype: F_2,61_ = 2.09; *p* = 0.132; pairwise comparisons for (+/+) and (+/−) with *p* = 0.104 and (+/+) and (−/−) with *p* = 0.070; 14-month-old mice: ANOVA, main effect for genotype: F_2,57_ = 5.5524; *p* = 0.006; pairwise comparisons for (+/+) and (+/−) with *p* = 0.038 and (+/+) and (−/−) with *p* = 0.002. (J) Percentage time of freezing at day 3, context B. Eight-month-old mice: ANOVA, main effect for genotype: F_2,61_ = 0.429; *p* = 0.653; pairwise comparisons for (+/+) and (+/−) with *p* = 0.565 and (+/+) and (−/−) with *p* = 0.716; 14-month-old mice: ANOVA, main effect for genotype: F_2,57_ *= 1*.*611*; *p* = 0.209; pairwise comparisons for (+/+) and (+/−) with *p* = 0.771 and (+/+) and (−/−) with *p* = 0.091. (K, L) Percentage time of freezing at days 4–7, context A, indicative of extinction learning in 8-month-old mice (K) and 14-month-old mice (L). Three-way ANOVA (day × genotype × age): F_6,354_ = 2.021; *p <* 0.062; 8-month-old mice: two-way ANOVA (day × genotype): F_6,183_ = 0.862; *p* = 0.524; main effect for genotype: F_2,61_ = 0.305; *p* = 0.738. Fourteen-month-old mice: two-way ANOVA (day × genotype): F_6,171_ = 2.845; *p* = 0.011; main effect for genotype: F_1,57_ = 35.484: *p <* 0.0001; pairwise comparisons (+/+) and (+/−): *p <* 0.0001 and for (+/+) and (−/−) *p <* 0.0001. Eight-month-old cohort: (+/+) M = 12 mice, (+/+) F = 11 mice; (+/−) M = 10 mice, (+/−) F = 9 mice; (−/−) M = 11 mice, (−/−) F = 11 mice. Fourteen-month-old cohort: (+/+) M = 11 mice, (+/+) F = 12 mice, (+/−) M = 10 mice, (+/−) F = 12 mice, (−/−) M = 9 mice, (−/−) F = 6 mice. *p < 0.05, **p < 0.01, ***p < 0.001. Data are represented as means ± SEM. Individual quantitative observations that underlie the data presented in this figure are summarized in S2 Data. F, female; M, male.

A recent study analyzing mutations in the human spastin gene reported patients with learning disabilities and memory impairments [8]. All patients with memory deficits were in the range of 46–77 years of age and carried missense mutations located in the highly conserved AAA domain, mediating ATPase function [8]. These findings prompted us to study cognitive aspects such as learning and memory in spastin knockout mice lacking gene expression of this microtubule-severing protein. In a Y-maze spontaneous alternation (SA) test, spastin depletion (−/−) resulted in reduced short-term spatial recognition memory at 8 and 14 months of age as compared with wild-type (+/+) littermate controls (Fig 2A and 2B). These deficits were already detectable in heterozygous mutants at 8 months of age, and they turned out to be independent of their overall exploration activity because total arm entries were comparable across genotypes (Fig 2C). A related T-maze experiment confirmed this memory deficit (Fig 2D and 2E), indicating that the absence of functional spastin indeed affects working and reference memory. To further examine cognitive function, we tested spastin knockout mice for context associative conditioned fear memory (Fig 2F). At day 1 of the experiment, spastin +/+, +/−, and −/− mice at 8 and 14 months of age displayed equal freezing responses (often used as a measure of conditioned fear in analyzing memory formation), suggesting normal acquisition of contextual fear memories (Fig 2G and 2H). Upon reexposure to the training context (context A on day 2), spastin-depleted mice at 14 months of age exhibited significantly lower freezing levels in both the heterozygous and homozygous condition, indicating that older animals had problems with memorizing the context–shock association (Fig 2I). In contrast, exposure to a different unrelated context (context B on day 3) led to equal freezing levels across genotypes and age (Fig 2J), indicating a context-specific formation of associative memories. Next, we tested both age groups in an extinction-learning paradigm, which refers to the gradual decrease in response to the conditioned stimulus that occurs when the stimulus (the context) is presented without reinforcement (reexposure to context A over 4 consecutive days without receiving a shock). This experiment led to decreased context-associated freezing levels, as shown for all genotypes of the younger cohort (Fig 2K), indicating a successful extinction-learning process. However, older animals revealed significant impairments in memory extinction for both +/− and −/− genotypes as compared with control mice (+/+) (Fig 2L). Interestingly, −/− mice not only revealed a lack of extinction (compare with +/− mice) but in addition displayed the highest freezing levels throughout all groups. Together, these data indicate that spastin-mediated functions are critical for working and associative memory in mice. Consistent with observations from human patients carrying SPG4 mutations, these memory deficits were detectable in homozygous and heterozygous genotypes and were mainly observed at advanced age. During their second half of life, the spastin knockout mouse therefore resembles some cognitive aspects of cHSP [8].

### Spastin depletion affects the number, structure, and function of hippocampal synapses

The structural and functional connectivity of neuronal networks, a cellular basis for cognitive performance, is highly vulnerable under neurodegenerative conditions [30]. Dynamic microtubules are key organizers of axons and dendrites. They deliver multiple intracellular cargoes involved in the formation and function of synaptic connections and invade dendritic spines in an activity-dependent manner [31–33].

**Fig 3:**
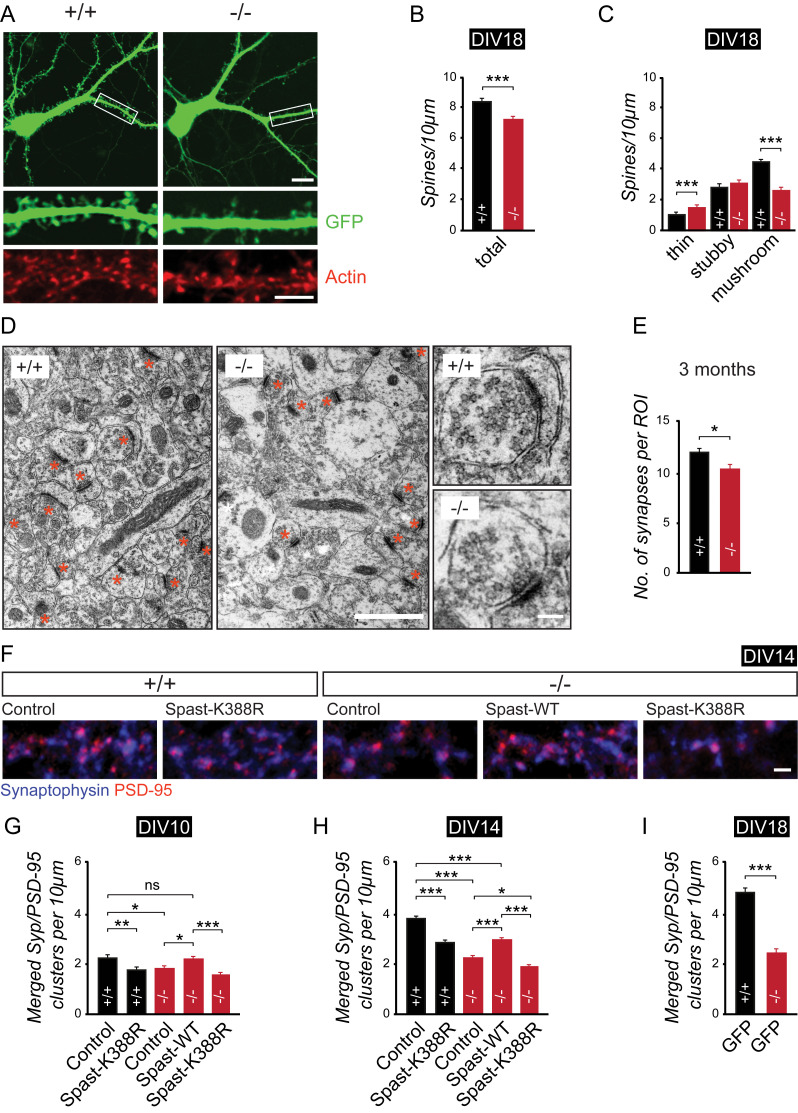
Spastin depletion reduces spine and synapse density. (A) Representative images of rhodamine-phalloidin–labeled neurons at DIV18. EGFP used as volume marker transfected at DIV10. (B) Quantification of total number of spines per 10 μm dendrite length. (+/+), *n* = 30; (−/−), *n* = 35. (C) Quantification of spine types per 10 μm dendrite length. Scale bar, 10 μm; magnification, scale bar, 5 μm. (+/+), *n* = 30; (−/−), *n* = 35. (D) EM micrographs of CA1 region from spastin (+/+) and (−/−) mice (3 months). *n* = 3 mice per group. Scale bar, 1 μm; magnification, scale bar, 100 nm. (E) Quantification of synapse numbers per ROI (18 μm^2^), (+/+), *n* = 17; (−/−), *n* = 21. (F) Coimmunostaining of Syp and PSD-95 at DIV14 to visualize excitatory synaptic contacts (magenta signals), following expression of different GFP-Spastin constructs as indicated. Representative dendritic regions of hippocampal neurons are depicted; scale bar, 2 μm. (G-I) Quantification of synapses per 10 μm dendrite length at DIV10, 14 and 18; *n* = 80–98 dendritic regions per group. *p < 0.05, **p < 0.01, ***p < 0.001. Student *t* test and ANOVA followed by pairwise comparison were used to assess statistical significance. Data are represented as means ± SEM. Individual quantitative observations that underlie the data presented in this figure are summarized in S3 Data. DIV, days in vitro; EGFP, enhanced green fluorescent protein; EM, electron microscopy; GFP, green fluorescent protein; ns, not significant; PSD-95, postsynaptic density protein 95; ROI, region of interest; Syp, synaptophysin.

Disturbed memory function may therefore stem from abnormalities in the morphology or physiology of synapses. To this end, we examined the structure of dendritic spines and analyzed the number of synaptic contacts. Quantitative assessment in primary hippocampal neurons derived from wild-type (+/+) and spastin knockout (−/−) mice revealed a significantly reduced spine density (Fig 3A and 3B). Notably, mushroom-type spines were mainly affected by the loss of spastin, whereas the number of thin and potentially immature spines turned out to be increased (Fig 3C). We confirmed these observations at the ultrastructural level by applying electron microscopy (EM). Also, in this experiment, loss of spastin gene expression (−/−) caused a significant reduction of asymmetric (spine) synapses in the CA1 region of hippocampal slices in the range of 20% (Fig 3D and 3E).

To test whether spastin depletion is the underlying cause of the observed deficits, we performed rescue experiments at different developmental stages. To this end, we reexpressed wild-type spastin or spastin-K388R on the genetic background of cultured primary hippocampal knockout neurons, the latter of which carries a mutation identified in human SPG4-type HSP [34] known to inhibit microtubule severing [10]. Colocalized signals of presynaptic synaptophysin and postsynaptic density protein 95 (PSD-95), representing glutamatergic synapses, were significantly reduced in knockout (−/−) neurons as compared with wild-type (+/+) cells (Fig 3F–3I). Notably, spastin reexpression rescued (days in vitro [DIV]10) or attenuated (DIV14) the loss of synapses, whereas the spastin-K388R mutant was not sufficient to compensate this effect (Fig 3F–3H). Under these conditions, expression of the microtubule-severing–deficient spastin-K388R mutant on spastin knockout background further reduced synapse density at DIV14 as compared with the simple loss of spastin. Our data therefore suggest that spastin-mediated microtubule severing represents a critical parameter in spine maturation and synapse formation and/or stability.

**Fig 4:**
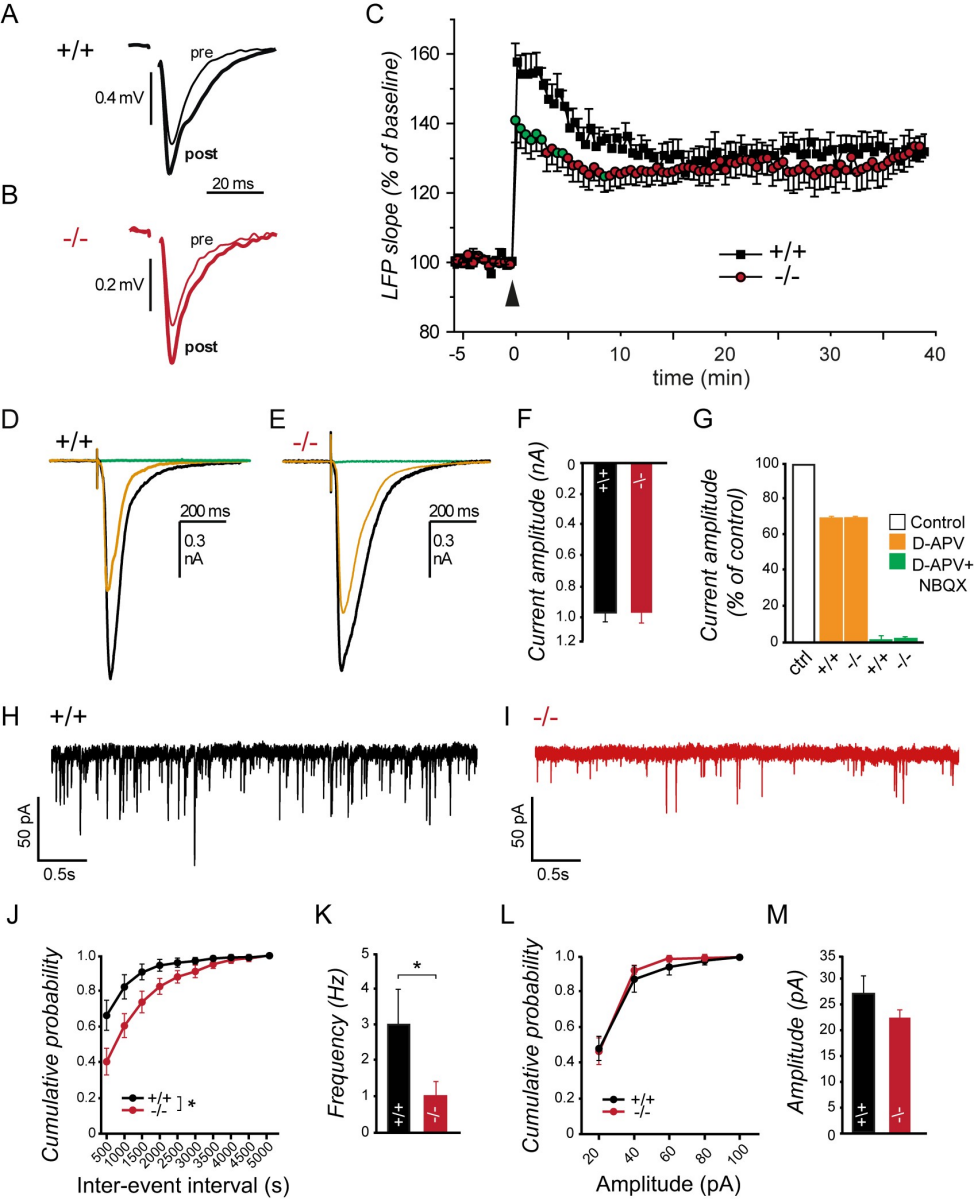
Spastin depletion affects LFPs and EPSC frequency. (A-C) LFPs upon single-pulse stimulation in CA1 before (pre) and 20 minutes after (post) theta burst stimulation (time point: 0 minutes, black arrow) of CA3 Schaffer collaterals in (+/+) and (−/−) mice. The gap in the recording traces results from elimination of the stimulation artifact immediately before the LFP response. Less pronounced increase in LFP slope in spastin-deficient mice (−/−, circles). Green circles indicate significantly different values (*p <* 0.05) during the first 5 minutes following stimulation (black arrow) compared with (+/+) littermates (filled squares). (+/+) *n* = 7, (−/−) *n* = 9. (D-F) EPSC amplitudes at mossy-fiber-to-CA3 pyramidal cell synapses using whole-cell patch-clamp recordings. Synaptic currents evoked by mossy-fiber stimulation in CA3 pyramidal cells. Black: controls; orange: 50 μM D-APV; green: D-APV plus 10 μM NBQX. (+/+) *n* = 8, (−/−) *n* = 9. (G) Equal D-APV–sensitive and D-APV–insensitive synaptic currents indicate unaltered contributions of NMDARs and AMPARs in both genotypes. (+/+) *n* = 7, (−/−) *n* = 5. (H, I) Whole-cell patch-clamp recordings using DIV14–18 neurons. (J-M) Cumulative probability histograms for mEPSC inter-event intervals and amplitudes. (+/+) *n* = 8, (−/−) *n* = 14. *p < 0.05. Student *t* test was used to assess statistical significance. Data are represented as means ± SEM. Individual quantitative observations that underlie the data presented in this figure are summarized in S4 Data. D-APV, d-2-amino-5-phosphonovaleric acid; DIV, days in vitro; EPSC, excitatory postsynaptic current; LFP, local field potential; mEPSC, miniature EPSC; NBQX, 2,3-dioxo-6-nitro-7-sulfamoyl-benzo[f]quinoxaline; NMDAR, *N*-methyl-d-aspartate receptor.

We therefore asked whether spastin depletion may affect local field potentials (LFPs) that reflect the postsynaptic population response of hippocampal Schaffer collateral-to-CA1 pyramidal cell synapses in the stratum radiatum. Theta burst stimulation (TBS) of acute brain slices derived from wild-type (+/+) and spastin-deficient (−/−) mice increased LFP amplitude and slope in both genotypes (Fig 4A–4C). In the absence of spastin, the increase in LFP slope was significantly less pronounced during the first 5 minutes following stimulation (Fig 4C, green data points). To assess the relative contributions of *N*-methyl-d-aspartate receptors (NMDARs) and AMPARs, we then electrically stimulated mossy-fiber-to-CA3 pyramidal cell synapses using acute brain slices and isolated AMPAR-mediated synaptic currents by blocking NMDARs with 50 μM d-2-amino-5-phosphonovaleric acid (D-APV). In this experiment, the ratio of D-APV–sensitive NMDAR-mediated currents and D-APV–insensitive AMPAR-mediated currents remained equal in slices derived from wild-type (+/+) and spastin knockout (−/−) animals (Fig 4D–4G), suggesting that despite a significant loss of synapse numbers (Fig 3), remaining synapses contain equal amounts of NMDARs and AMPARs in spastin knockout animals compared with wild-type mice. A subsequent spontaneous miniature excitatory postsynaptic current (mEPSC) analysis using cultured hippocampal neurons confirmed equal amplitudes of AMPAR currents in neurons from both genotypes but revealed a significant decrease in current frequencies by more than 50% (Fig 4H–4M). In summary of the morphological and physiological data, we conclude that spastin depletion reduces synapse number, thereby affecting synaptic connectivity.

### Altered microtubule structure and dynamics in the absence of spastin gene expression

**Fig 5:**
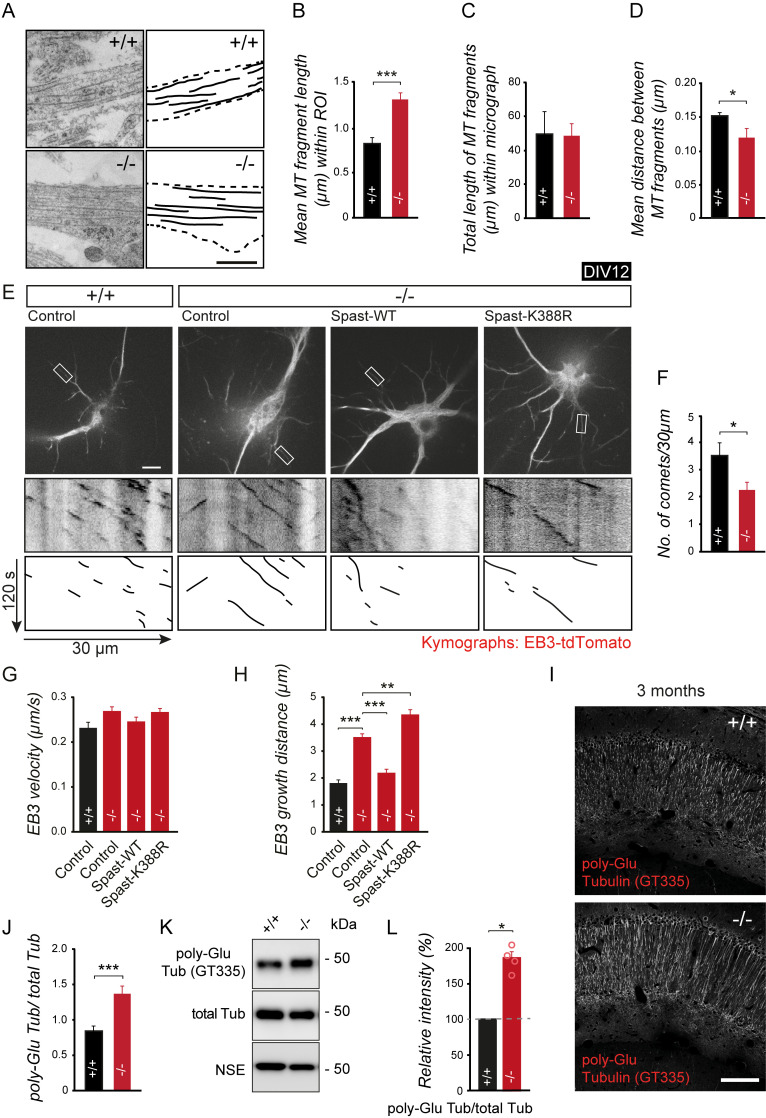
Spastin depletion alters growth and structure of MTs. (A) Electron micrograph of MT in DIV14 neurons. Note that because of ultrathin sectioning, MTs appear as fragments in three-dimensional dendrites growing across other neurites. Scale bar, 200 nm. (B-D) Quantification of MT mean fragment length, total fragment length, and inter-microtubule fragment distance. (+/+) *n* = 20, (−/−) *n* = 14 based on three independent neuronal cultures per group. (E-H) Live imaging of EB3-tdTomato in DIV12 neurons following coexpression of different GFP-Spastin constructs, as indicated. (E) Upper panel depicts GFP expression. Lower panels: representative kymographs of EB3-tdTomato acquired from secondary dendrites (white rectangle). Scale bar, 10 μm. Quantification of (F) comet number, (G) velocity, and (H) growth distance; (+/+_GFP) *n* = 36 (12 cells), (−/−_GFP) *n* = 80 (27 cells), (−/−_Spast-WT) *n* = 42 (14 cells), (−/−_Spast-K388R) *n* = 48 (16 cells). (I) Immunostaining of polyglutamylated tubulin (GT335 antibody) in CA1. (+/+), *n* = 9 mice; (−/−), *n* = 9 mice. Scale bar, 100 μm. (J) Quantification of (I). (K, L) Quantitative western blot analysis of Poly-Glu Tub, total α-Tub, and NSE (loading control). (+/+) *n* = 4, (−/−) *n* = 4, **p < 0.01, ***p < 0.001. Student *t* test (B-D and J), ANOVA followed by pairwise comparison (F-H), and one-way ANOVA (L) were used to assess statistical significance. Data are represented as means ± SEM. Individual quantitative observations that underlie the data presented in this figure are summarized in S5 Data. DIV, days in vitro; EB3, end-binding protein 3; GFP, green fluorescent protein MT, microtubule; NSE, neuron specific enolase; Poly-Glu, polyglutamylation; PTM, posttranslational modification; ROI, region of interest; Tub, tubulin; WT, wild-type.

These results prompted us to examine the microtubule cytoskeleton, which provides the tracks for the delivery of various subcellular cargoes and is the direct target of spastin-mediated microtubule severing. At the EM level, ultrathin slices of neuronal dendrites derived from spastin knockout (−/−) mice exhibited significantly longer microtubule fragments with a significantly shorter distance to each other, pointing to long microtubules with an increased packing density (Fig 5A–5D). Live cell imaging using a fluorescent microtubule +TIP binding protein (EB3) further revealed that the lack of spastin affects microtubule growth. Using spastin −/− neurons, we observed a significantly reduced number of mobile EB3-tdTomato comets in secondary dendrites (in the range of 40%) as compared with wild-type (+/+) controls (Fig 5E and 5F). Interestingly, microtubules in knockout (−/−) neurons turned out to grow with a similar velocity but over longer distances (Fig 5G and 5H). Depletion of spastin and consequently less microtubule severing therefore compromises microtubule dynamics [9]. Rescue experiments with wild-type spastin, but not the spastin-K388R mutant on knockout background, normalized these effects. Notably, in comparison to the loss of spastin, expression of spastin-K388R further increased microtubule growth distance (Fig 5H), indicating that spastin’s microtubule-severing activity is causally involved in microtubule growth regulation.

Microtubules undergo PTMs [35, 36], known to alter microtubule function. For instance, the addition of polyglutamyl side chains to the C-terminal tail of tubulins, known as polyglutamylation, adds negative charge to the microtubule surface and regulates the binding of microtubule-associated proteins (MAPs) [37]. Previous studies further showed that efficient spastin-mediated microtubule severing depends on a critical number of glutamate molecules attached to the C-terminal tubulin tail [38, 39]. We therefore asked whether microtubule polyglutamylation levels in the hippocampal CA1 region may be affected upon spastin depletion. Indeed, immunostaining of hippocampal slices with a polyglutamylation-specific antibody revealed significantly increased tubulin polyglutamylation levels in spastin knockout (−/−) mice as normalized to total tubulin (Fig 5I and 5J and S3A and S3B Fig). Western blot analysis using hippocampal lysates derived from (+/+) and (−/−) animals confirmed this result (Fig 5K and 5L). We therefore conclude that the absence of spastin and consequently the lack of spastin-mediated microtubule severing accumulates polyglutamyl side chains on tubulin, leading to microtubule hyperglutamylation in spastin-depleted neurons.

### Loss of spastin alters kinesin-mediated transport and affects the delivery of synaptic vesicles and proteins

**Fig 6:**
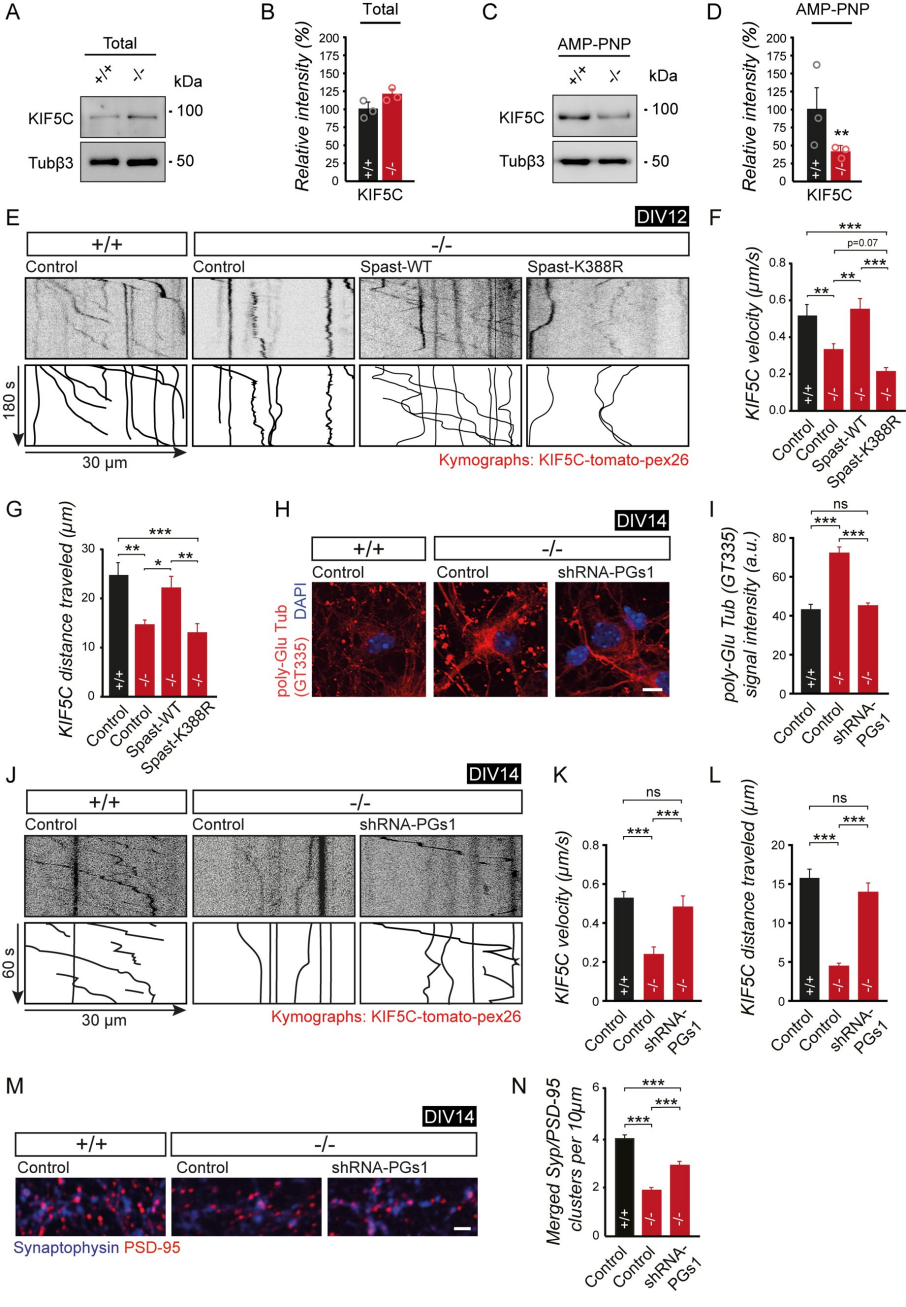
Impaired kinesin processivity is rescued by reducing tubulin polyglutamylation in spastin KO neurons. (A) Representative western blot depicting total KIF5C expression levels from brain homogenate of spastin (+/+) and (−/−) mice. (B) Quantification of data shown in (A). (C) Representative western blot depicting binding of KIF5C to MTs from brain homogenate of spastin (+/+) and (−/−) mice in the presence of nonhydrolyzable AMP-PNP (promotes kinesin–MT binding). (D) Quantification of (C). Data were normalized to Tubβ3. The percentage of the mutant signal relative to the WT signal is plotted. (+/+) *n* = 3, (−/−) *n* = 3 (MT preparations from independent mouse brains). One-way ANOVA was used to assess statistical significance. (E-G) KIF5C motility in dendrites at DIV12 using KIF5C without a cargo-binding domain fused to tdTomato and pex26 (peroxisome binding domain) KIF5C-tdTomato-pex26 is coexpressed with different GFP-Spastin constructs as indicated. (E) Representative kymographs of KIF5C. (F) Quantification of mean KIF5C velocity (G) Quantification of mean traveled distances; (+/+_control) *n* = 60 (20 cells), (−/−_control) *n* = 51 (17 cells), (−/−_Spast-WT) *n* = 59 (20 cells), (−/−_Spast-K388R) *n* = 51 (17 cells). (H) Representative immunostaining of polyglutamylated tubulin (GT335 antibody) using (+/+) or (−/−) DIV14 hippocampal neurons 3 days after transfection of control or shRNA-PGs1 constructs. Scale bar, 10 μm. (I) Quantification of GT335 signal intensities as shown in (H) (+/+_control) *n* = 26 cells, (−/−_control) *n* = 46, (−/−_shRNA-PGs1) *n* = 46. (J-L) KIF5C-tdTomato-pex26 motility in dendrites at DIV14 following cotransfection of control or shRNA-PGs1 constructs, both encoding a GFP reporter (hippocampal neurons derived from spastin [+/+] and [−/−] mice). (J) Representative kymographs of KIF5C. (K) Quantification of mean KIF5C velocity. (L) Quantification of mean travel distances; (+/+_control) *n* = 40 (11 cells), (−/−_control) *n* = 50 (18 cells), (−/−_shRNA-PGs1) *n* = 45 (16 cells). Time-lapse images were taken at 0.5 frames per second. (M) Coimmunostaining of Syp and PSD-95 at DIV14 to visualize excitatory synaptic contacts (magenta signals) following cotransfection of control or shRNA-PGs1 constructs, both encoding a GFP reporter (hippocampal neurons derived from spastin [+/+] and [−/−] mice). Representative dendritic regions of hippocampal neurons are depicted; scale bar, 2 μm. (N) Quantification of synapses per 10 μm dendrite length as shown in (M); (+/+_control) *n* = 56 (28 cells), (−/−_control) *n* = 65 (33 cells), (−/−_shRNA-PGs1) *n* = 64 (32 cells). *p < 0.05, **p < 0.01, ***p < 0.001. Student *t* test and ANOVA followed by pairwise comparison were used to assess statistical significance. Data are represented as means ± SEM. Individual quantitative observations that underlie the data presented in this figure are summarized in S6 Data. AMP-PNP, adenylyl-imidodiphosphate; DIV, days in vitro; GFP, green fluorescent protein; KIF, kinesin family protein; KO, knockout; MT, microtubule; ns, not significant; pex26, peroxisome assembly protein 26; PGs1, polyglutamylase subunit 1; PSD-95, postsynaptic density protein 95; shRNA, short hairpin RNA; Syp, synaptophysin; Tubβ3, tubulin β3; WT, wild-type.

We therefore aimed to assess whether spastin depletion, resulting in tubulin hyperglutamylation, may influence the binding of motor proteins to microtubules and consequently alter neuronal transport. To this end, we applied the ATP analogue adenylyl-imidodiphosphate (AMP-PNP) to stabilize motor protein–microtubule interactions in a cosedimentation assay. An initial analysis indicated that the total protein expression level of the kinesin family member KIF5C remained equal in brain lysates of spastin-depleted as compared with wild-type mice (Fig 6A and 6B). Notably, using spastin knockout (−/−) brains, we observed significantly reduced cosedimentation of KIF5C, a driver of pre- and postsynaptic cargoes in axons and dendrites, respectively [17, 22–25, 40, 41] (Fig 6C and 6D). These data suggest reduced kinesin–microtubule binding affinities in the absence of spastin.

Accordingly, using a functional transport assay, KIF5C-tdTomato-peroxisome assembly protein 26 (pex26) fusion proteins displayed significantly reduced transport velocities and travel distances upon depletion of spastin gene expression in (−/−) cultured primary hippocampal neurons as compared with wild-type (+/+) controls (Fig 6E–6G). In a rescue approach, spastin reexpression on knockout background, but not expression of the microtubule-severing–deficient spastin-K388R mutant, normalized both transport parameters (Fig 6E–6G).

To find a mechanistic link between increased tubulin glutamylation (Fig 5I–5L) and KIF5 processivity observed in spastin knockout (−/−) mice (Fig 6E–6G), we applied a knockdown approach of polyglutamylase subunit 1 (PGs1), one of the major subunits of the tubulin tyrosin ligase like 1 (TTLL1), catalyzing 80% of neuronal tubulin polyglutamylation [42]. As previously shown, shRNA-mediated knockdown of PGs1 gene expression significantly reduces tubulin polyglutamylation in neurons [40]. Expression of shRNA-PGs1 over 3 days in spastin (−/−) cultured hippocampal neurons normalized the increase in tubulin polyglutamylation observed in knockout control neurons as compared with (+/+)-derived control-treated neurons at DIV14 (Fig 6H and 6I). Strikingly, these conditions further normalized the KIF5C transport deficits (Fig 6J–6L) and attenuated the loss of excitatory synapses (Fig 6M and 6N). We therefore conclude a functional relation between spastin-mediated severing of microtubules and tubulin polyglutamylation, which in turn regulates kinesin motor processivity underlying synapse integrity.

**Fig 7:**
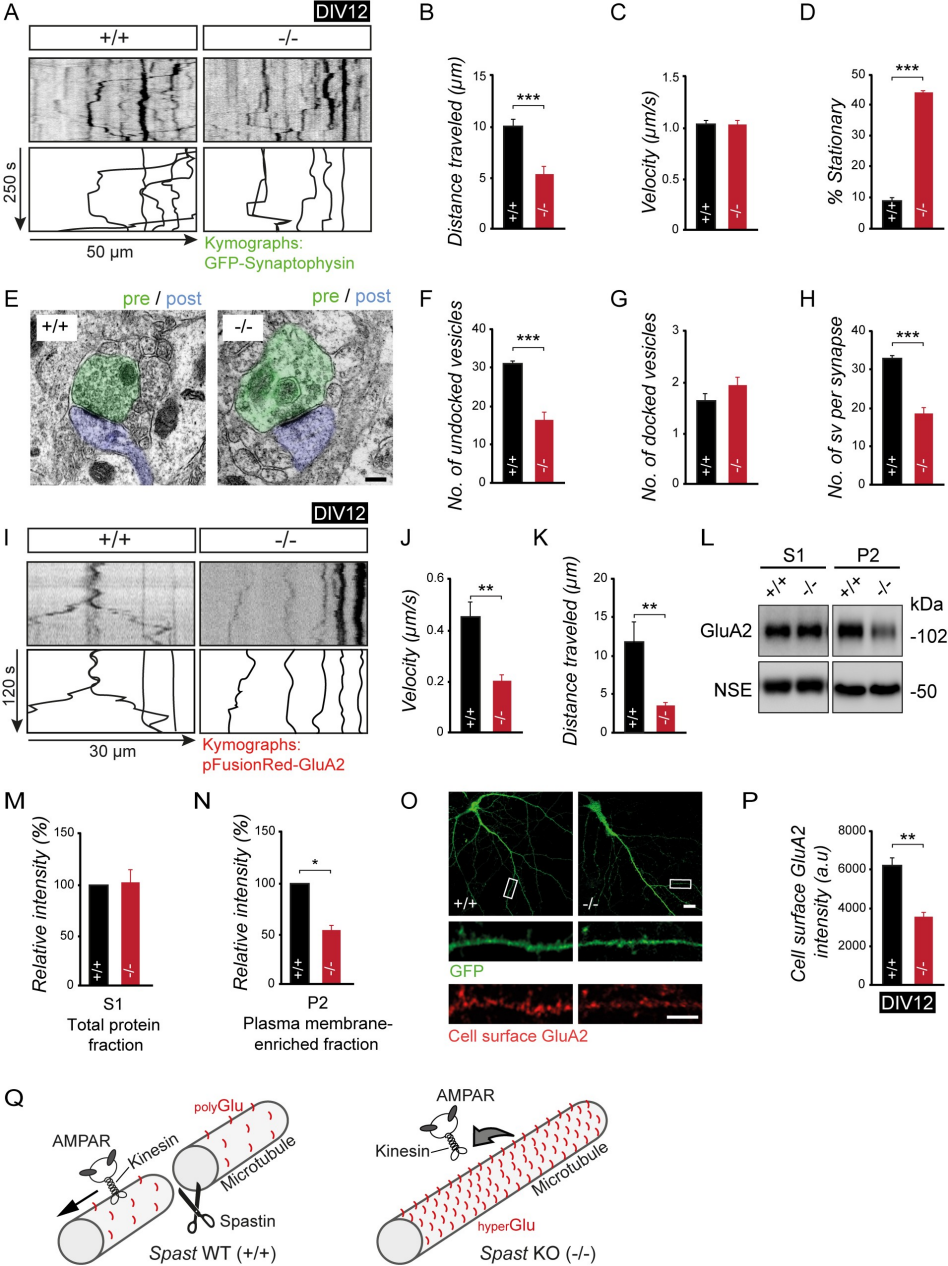
Spastin depletion impairs synaptic cargo transport and cell surface delivery. (A-D) Live imaging of Synaptophysin-EGFP transport. (A) Kymograph. (B) Travel distance. (C) Velocity. (D) Percent of stationary particles in axons. (+/+) *n* = 152 (15 cells), (−/−) *n* = 130 (12 cells). Images were taken at one frame per second. (E-H) Presynaptic vesicle analysis using EM. (E) Electron micrograph from CA1. Scale bar, 200 nm. (F) Undocked vesicle number. (G) Docked vesicle number. (H) Vesicles per synapse terminal. (+/+) *n* = 3 mice, (−/−) *n* = 3 mice. Student *t* test was used to assess statistical significance. (I-K) AMPAR neuronal transport detecting pFusionRed-GluA2 particle mobility over time. (I) Kymograph. (J) Velocity. (K) Travel distance. (+/+), *n* = 17 (12 cells) particles; (−/−), *n* = 16 (12 cells). (L-N) GluA2 subcellular expression levels analyzed using western blotting, following subcellular fractionation. (L) Representative western blots. (M) Quantification of GluA2 in total protein fraction (S1). (+/+), *n* = 7; (−/−), *n* = 6. (N) Quantification of GluA2 in plasma membrane–enriched fraction (P2). (+/+) *n* = 4, (−/−) *n* = 4. NSE used as loading control. (O, P) GluA2-AMPAR cell surface staining using DIV18 neurons. (O) Cell surface GluA2 (red) expression in dendrites; scale bar, 10 μm; magnification, 5 μm. EGFP (green) used as volume marker. (P) Cell surface GluA2 signal intensity. (+/+), *n* = 19; (−/−), *n* = 19. Student *t* test was used to assess statistical significance. *p < 0.05, **p < 0.01, ***p < 0.001. Data are represented as means ± SEM. (Q) Model. Loss of spastin-mediated MT severing leads to longer MTs characterized by an accumulation of tubulin polyglutamyl side chains. The resulting increase in negative charge at the MT surface affects the binding and mobility of motors (e.g., kinesins) and their respective cargo proteins (e.g., vesicular AMPARs). The observed deficits in synaptic cargo delivery are in agreement with the cognitive deficits in spastin KO mice and patients with SPG4-type HSP. Individual quantitative observations that underlie the data presented in this figure are summarized in S7 Data. AMPAR, α-amino-3-hydroxy-5-methyl-4-isoxazolepropionic acid receptor; DIV, days in vitro; EGFP, enhanced green fluorescent protein; EM, electron microscopy; GluA2, Glutamate receptor AMPA type subunit 2; KO, knockout; MT, microtubule; NSE, neuron specific enolase; SPG, spastic paraplegia.

Finally, we asked whether neurons derived from spastin knockout (−/−) mice may be characterized by an abnormal delivery and distribution of synaptic cargoes. With respect to kinesin-dependent trafficking of synaptic vesicles and proteins [17, 22–25, 40, 41], we observed reduced travel distances of the presynaptic vesicle marker synaptophysin in spastin knockout (−/−) neurons, while its velocity remained unchanged (Fig 7A–7C). These findings were accompanied by increased numbers of stationary particles under spastin-depleted conditions (−/−) (Fig 7A and 7D). Accordingly, at the ultrastructural level, the numbers of detectable presynaptic vesicles at axon terminal boutons turned out to be significantly reduced (Fig 7E–7H), which may contribute to the reduced mEPSC frequencies observed in this study (compare with Fig 4H–4K).

Likewise, the dendritic mobility parameters of KIF5-dependent postsynaptic GluA2 subunit–containing AMPARs were markedly reduced upon spastin depletion (Fig 7I–7K), a finding which correlated with reduced amounts of GluA2-type AMPARs in plasma membrane–enriched brain lysate derived from spastin knockout (−/−) mice (Fig 7L–7N). The analysis of GluA2 fluorescence intensities, cluster numbers, and cluster sizes confirmed this view, showing that AMPAR levels across the overall neuronal cell surface were significantly diminished (Fig 7O and 7P and S4 Fig).

Collectively, our data provide the first evidence that the loss of spastin gene expression, known to affect spastin-mediated microtubule severing [12], regulates kinesin processivity controlled by tubulin polyglutamylation (Fig 7Q). The observed deficits in synaptic cargo delivery and synaptic transmission are in agreement with cognitive deficits in spastin knockout mice and patients with cHSP.

## Discussion

The present study aimed to elucidate mechanistic insight into the functional role of spastin-mediated microtubule severing in neurons. Complicated forms of SPG4-type HSP (cHSP), characterized by mutations in the spastin gene, cause deficits in motor and cognitive performance; however, the underlying subcellular mechanisms that lead to neuronal dysfunction have remained elusive. Using heterozygous and homozygous spastin knockout mice, we confirmed the previously known deficits in motor performance (Fig 1) and for the first time observed cognitive impairments that show some similarity with cHSP phenotypes. Our data reveal that spastin regulates working and associative memory (Fig 2), likely due to specific subcellular deficits in neurons of the CNS. At the molecular level, our study provides mechanistic insights indicating that spastin depletion (1) reduces synapse numbers and excitatory postsynaptic current (EPSC) frequencies (Figs 3 and 4); (2) increases microtubule growth distance and causes longer, hyperglutamylated microtubules (Fig 5); (3) interferes with kinesin–microtubule binding efficiency and motor protein processivity based on altered tubulin polyglutamylation levels (Fig 6); and (4) leads to transport deficits of synaptic vesicles and AMPARs (Fig 7). These results are specific to the loss of spastin, since rescue approaches through spastin reexpression on the genetic knockout background normalized the observed functional deficits at the (1) synaptic, (2) microtubule, and (3) transport level. Notably, reexpression of the microtubule-severing–deficient spastin mutant K388R, identified in human SPG4 patients, failed to rescue any of these deficits. Furthermore, a knockdown of PGs1, a major subunit of the main tubulin glutamylase in brain, not only normalized the increase in tubulin polyglutamylation following spastin depletion in neurons but also rescued transport deficits and attenuated the loss of synapse numbers. This suggests that the enhancement of tubulin polyglutamylation is likely the cause of kinesin-mediated transport defects.

It is plausible to conclude that spastin-mediated changes in microtubule severing and growth regulate motor-cargo transport and contribute to the cognitive deficits of spastin knockout mice and possibly SPG4 patients. Interestingly, besides mutations in a variety of genes, HSP can be due to mutations in the genes encoding spastin (SPG4) and KIF5A (SPG10), both of which represent proteins that functionally associate with microtubules. Our study is consistent with the interpretation that spastin dysfunction acts upstream of kinesin dysfunction; however, we cannot rule out that altered kinesin transport causes conditions that in turn affect spastin severing activity.

The most prominent phenotype of HSP, including SPG4-type HSP, is characterized by neurodegeneration in the corticospinal tracts, leading to lower limb spasticity and motor deficits [9]. In contrast, some patients, often at increased age, develop complicated forms of HSP that are characterized by cognitive impairment and dementia accompanied by psychiatric comorbidities [7, 8, 43, 44]. Clinical findings reported cognitive deficits primarily in the second half of life, typically from 40–50 years of age onward [43, 44]. These observations are consistent with our data, which have revealed that spastin knockout mice display their major memory and extinction deficits at 14 but not 8 months of age. Notably, homozygous (−/−) spastin knockout mice not only failed to extinguish contextual fear memory (Fig 2L) but in general showed increased freezing responses, which may be explained by an overactive time-dependent “incubation” of fear memory [45]. Furthermore, it is striking that previous studies connected conditioning and consolidation of fear memories with a biphasic shift of labile and stable microtubule fractions [33, 46]. Because spastin severing promotes the generation of new microtubule fragments [9], spastin depletion likely unbalances the distribution of these fractions, thereby affecting cytoskeletal rearrangement during cognitive processes. However, in contrast to the memory deficits observed upon spastin depletion, it is noteworthy that spastin seems not to be involved in the mechanisms underlying the acquisition of contextual fear memory because haploinsufficient and homozygous spastin knockout animals acquired the task comparably to wild-type +/+ controls (Fig 2G and 2H).

Microtubule dynamics has been implicated as an important factor in learning and memory and may be compromised in neurodegenerative diseases such as Alzheimer disease [33, 46]. It remains, however, barely understood which subcellular mechanisms bridge a dynamic microtubule cytoskeleton with synaptic transmission or plasticity and which proteins are the most critical factors in this respect. A recent study showed a role of the tubulin binding protein stathmin, a regulator of microtubule polymerization, in controlling neurogenesis, spinogenesis, and synaptic NMDAR levels. Stat4A mutant mice consequently exhibited deficits in hippocampus-dependent fear conditioning [47]. With respect to cognitive function, it is unknown whether microtubule growth, microtubule–kinesin binding, and motor-cargo transport contribute to synaptic function and connectivity. Our study suggests that disturbing normal microtubule severing interferes with the delivery of different intracellular cargoes, thereby reducing synapse numbers and neuronal function. It is plausible to conclude that reduced presynaptic vesicles and reduced mushroom-type spine synapses may underlie the working and associative memory deficits observed in this study. Although this conclusion is based on correlation, human patients with SPG4 (spastin encoding gene) develop intellectual disabilities and late-onset dementia. In agreement with a recent hypothesis [48], some microtubule phenotypes observed in our study may be due to a toxic gain-of-function effect of the spastin-K388R mutant (Figs 3, 5 and 6) [48].

At the molecular level, it is suggested that reduced spastin activity in patients with SPG4-type HSP may be sufficient to cause longer and less dynamic microtubules [9]. Accordingly, using EB3 imaging and EM in CNS neurons lacking spastin gene expression, our data confirm this view. It is presently speculative whether microtubule growth parameters themselves participate in the reduced binding affinity between microtubules and kinesin motors observed in our study. More likely, tubulin polyglutamylation accumulates upon the loss of spastin-mediated severing, thereby increasing the negative charge at the microtubule surface, which in turn interferes with efficient kinesin binding [49] (compare with Fig 5I–5L, Fig 6C and 6D and Fig 7Q). Alternatively, hyperglutamylation could mainly affect binding of MAPs, which may indirectly regulate kinesin binding to microtubules [37, 50, 51]. Although this requires further investigation, reduced microtubule severing seems to be critical in shifting the equilibrium of tubulin polyglutamylation levels toward higher values.

It has remained controversial whether tubulin polyglutamylation promotes or inhibits motor transport. Whereas in vitro assays revealed that kinesin motility on neuronal β3-tubulin (TUBB3) is increased by polyglutamylation [52], cellular studies in neurons observed that increased polyglutamylation (following neuronal activation or double knockout of two tubulin deglutamylases) correlated with reduced cargo transport [41, 53, 54]. Consistent with the latter observation, hyperglutamylated microtubules in the present study confirm reduced kinesin (KIF5) binding affinity in the presence of the ATP analogue AMP-PNP (Fig 6C and 6D) using spastin-depleted neurons. Remarkably, Larcher and colleagues reported that kinesin affinity to polyglutamylated microtubules depends on the actual tubulin subunit and the length of the individual glutamyl side chain [51], indicating that polyglutamylation is a regulatory component in kinesin transport. In line with this, normalizing tubulin polyglutamylation levels led to a rescue of transport deficits in spastin knockout neurons in our study (Fig 6J–6L). Together, we conclude that kinesin-track binding seems to be a highly regulated process that is sensitive to glutamyl side chains at C-terminal tubulin tails.

KIF5 delivers AMPARs and various synaptic cargoes into dendrites and axons [17, 22–25, 40, 41]. Diminished kinesin–microtubule binding controlled by tubulin polyglutamylation, as observed through spastin depletion in this study, may therefore be the underlying cause that presynaptic vesicles and AMPARs levels are not properly delivered to axon terminals or the dendritic cell surface, respectively. Likewise, the synaptic deficits observed by our electrophysiological analysis are likely to depend on the reduction of these synaptic cargoes, although we cannot rule out that other delivery problems contribute to the phenotype.

It is however notable that although there is a reduction in cell surface AMPARs in spastin (−/−) neurons, individual synapses display similar mEPSC amplitudes as compared with wild-type control neurons, indicating equal AMPAR content at the postsynaptic specialization. This phenomenon is not unexpected, because the pool of extrasynaptic AMPARs, which represents a reserve pool for LTP [55–57], is much larger than the number of synaptic AMPARs. We therefore conclude that although we observe a reduction of AMPARs at the overall plasma membrane, this is not sufficient to alter AMPAR levels at the remaining synapses in spastin knockout neurons. This interpretation is consistent with the results following genetic depletion of the microtubule growth regulator KIF21B [32]. It further supports the view that plasma membrane AMPARs diffuse laterally from extrasynaptic reserve pools and travel independently of the microtubule cytoskeleton to reach their final postsynaptic destination [55–57].

In summary, the present study provides the first evidence that the loss of spastin resembles cognitive deficits of patients with SPG4-type HSP and suggests that they depend on synaptic changes in the CNS. Our data provide a potential mechanism of how impaired spastin-mediated microtubule severing accumulates polyglutamylation levels and consequently affects kinesin binding and cargo transport. The spastin knockout mouse may therefore be suitable to develop new treatment strategies against HSP.

## Materials and methods

### Ethics statement

All animal experiments were in strict accordance with the principles of laboratory animal care (NIH publication No. 86–23, revised 1985) and the recommendations in the Guide for the Care and Use of Laboratory Animals of the German Animal Welfare Act on protection of animals. All behavioral tests were approved by German and European laws as well as by local authorities (Authority of Soziales, Familie, Gesundheit und Verbraucherschutz, Committee for Lebensmittelsicherheit und Verbraucherschutz, Billstraße 80, 20539 Hamburg, Germany, reference [ID number] 100/13). Generation of spastin knockout mice (Spast^−/−^) was previously described [28].

### DNA constructs

The following previously available vectors were used in the study: GFP-C1 (Clontech #632470, Kusatsu, Japan), Synaptophysin-EGFP [59] (Addgene, Cat#73816), pCl-fusionRed-GluA2 [60] (Addgene, Cat#24001), and EGFP-Spastin-WT [61].

EB3-tdTomato was generated by subcloning of EB3 from EB3-EGFP (Anna Akhmanova, Faculty of Science, Utrecht, The Netherlands) into tdTomato-N1 (Clonetech) using EcoRI/BamHI. To generate KIF5c-tdTomato-pex26, the KIF5c motor domain, containing amino acid 1–370, was subcloned into the expression vector pcDNA3 using HindIII/EcoRI. Subsequently, tdTomato was inserted using EcoRI/XhoI. Finally, pex26 [58] was inserted using XhoI/ApaI. EGFP-Spastin-K388R was generated by site-directed mutagenesis based on the Spastin-WT backbone. The nucleotide exchange of *Spast*_A1288G was generated using the following oligonucleotides: LR_FwiSpast, CTATAACGAGAGAGTACTAACCTGACATGC; LR_RviSpast, GCGGTCAGATCACTTCCAGAGTATC; and PB-DNSpast-KR_s, CAGGAAACGGAAgAACAATGCTGGC. This resulted in the microtubule-severing–deficient Spastin-K388R mutant [34]. To knock down PGs1, required for TTLL1-mediated polyglutamylation of tubulin, shRNA-PGs1 or a control vector, both encoding GFP as a reporter, were used [41, 42].

### Microtubule binding assay

To determine whether the lack of spastin could affect the binding of motor proteins to the microtubules, we performed a microtubule cosedimentation assay using a modified version of a protocol previously reported [62]. In brief, hippocampi were homogenized in PIPES buffer (1 mM EGTA/100 mM PIPES, pH 6.8/1mM MgSO_4_) containing protease inhibitors (10 μM PMSF/5 μg/ml E64). The obtained lysates containing the crude tubulin were centrifuged at 50,000*g* (Beckman Coulter, Optima Max-XP, Brea, CA, USA) for 30 minutes at 4°C. The pellet was discarded, and 1 mM GTP and 20% glycerol were added to the remaining supernatant. This lysate comprises the microtubule-bound and the microtubule-free fractions and was referred to as the total fraction. The supernatant was equally distributed among two new tubes, and either 1 mM ATP (pH 6.8) or 1 mM AMP-PNP (pH 6.8) was added to each tube. After an incubation for 35 minutes at 37°C, the supernatant was treated with 20 μM Taxol. The mixture was centrifuged at 150,000*g* for 40 minutes at 37°C. The pellet was resuspended in 8 M urea and used for western blotting analysis.

### Live cell imaging

For time-lapse imaging, coverslips with the transfected neurons were mounted into a live cell imaging chamber containing conditioned medium and kept at 37°C and 5% CO_2_ levels in an incubator coupled to the spinning disk microscope. To observe the transport of GluA2, KIF5c, and synaptophysin clusters, neurons at DIV11 were transfected with respective constructs and analyzed 24 hours later. In order to examine microtubule dynamics, neurons at DIV11 were transfected with the microtubule plus-end marker EB3-tdTomato and imaged 24 hours later. Only EB3 comets that appeared and disappeared during a time period of 300 seconds (frame rate: 0.5 frames/second) were included in the analysis. For rescue experiments, neurons at DIV11 were cotransfected with KIF5c-tdTomato-pex26 and shRNA-PGs1 or a control plasmid, respectively, and imaged after 3 days. Time-lapse images of transfected neurons were acquired using a Nikon spinning disc microscope (Visitron, Puchheim, Germany) equipped with a 60× and 100× objective and 488-nm and 561-nm argon lasers. For the final analysis, captured LSM images were exported as TIF images. Anterograde and retrograde GluA2, KIF5c single particle mobility, as well as EB3-tdTomato comet growth of sequential images within secondary dendrites identified based on their morphology (presence of mushroom-type protrusions) were quantified manually using ImageJ software (ImageJ, NIH, USA). Background correction was achieved through subtraction of average intensity. Kymographs of 30-μm (GluA2, KIF5c, and EB3) or 50-μm (synaptophysin) dendritic segments were generated using the Multiple Kymograph plug-in for Fiji (ImageJ, NIH, USA). All the immunofluorescence quantifications were performed on the MetaMorph 6.3 r7 software (Molecular Devices, Sunnyvale, CA, USA) or using ImageJ. Coimmunostaining of both pre- and postsynaptic markers was quantified by thresholding the signal and applying a separation filter to isolate the separated puncta. Further, the number and fluorescent intensity of the puncta per region of interest (ROI) were measured. For surface receptor staining, ROIs were drawn manually in a given channel and superimposed on the complementary channel. Student *t* test was used for statistical analysis unless stated otherwise and was carried out using SPSS (PASW Statistics 18.0, IBM, New York, USA).

### Electrophysiological recordings

#### Whole-cell patch-clamp recordings

Whole-cell patch-clamp recordings were obtained from DIV18–20 hippocampal neurons. Recordings were made with 2.0–3.5 MΩ borosilicate pipettes filled with intracellular solution (120 mM K-gluconate, 8 mM NaCl, 2 mM MgCl_2_, 0.5 mM CaCl_2_, 5 mM EGTA, 10 mM HEPES, 14 mM phosphocreatine, 2 mM magnesium-ATP, 0.3 mM sodium-GTP, pH adjusted to 7.3 with KOH). Patchmaster software (HEKA, Lambrecht, Germany) in combination with an EPC 9 patch-clamp amplifier (HEKA) was used for stimulation and data acquisition. Recordings were low-pass filtered at 2.9 kHz and analyzed with Fitmaster (HEKA), Igor Pro 6.03 (Wavemetrics), Mini Analysis (Synaptosoft, Decatur, GA, USA), and Excel (Microsoft). Only neurons with an access resistance <20 MΩ were evaluated. mEPSCs were recorded from hippocampal neurons of +/+ (*n* = 8; 1,617 events) and spastin −/− neurons (*n* = 14; 1,471 events) for 30 seconds and analyzed with Mini Analysis software (Synaptosoft). Recordings were made at room temperature (21–23°C) in Ringer’s solution (143 mM NaCl, 5 mM 1 KCl, 0.8 mM MgCl_2_, 1 mM CaCl_2_, 10 mM HEPES, 5 mM glucose, and pH adjusted to 7.3 with NaOH). mEPSCs were recorded in the presence of TTX (0.25 μM), bicuculline (10 μM), and APV (20 μM), which were added to the control solution. All substances were purchased from Sigma-Aldrich.

### Behavioral analysis

#### Contextual fear conditioning

Fear conditioning is carried out in fear-conditioning chambers equipped with shock grid floor and digital NIR video fear-conditioning system (MED-VFC-SCT-M, Med Associates Inc, VT, USA) connected to a controller computer. Mice from each genotype and sex were examined in four successive phases comprising (1) conditioned acquisition (day 1), (2) memory for the conditioned background context A (day 2), (3) exploration in new background context B (day 3), and (4) extinction in the conditioned context A (days 4–7). Context A was characterized by a cubic shape, illuminated by 135 Lux, and the presence of a vanilla odor. The floor consisted of metal rods, mediating the foot shock. Context B was characterized by a pyramidal shape, not illuminated, and without additional odor cues. The background noise for context A and B was 65 dB. On day 1, mice were introduced to the conditioning chambers scented with vanilla odor (RUF Lebensmittelwerk, Quakenbruck, Germany) for 8 minutes and received three unconditioned stimuli (US: 1 second, 0.25 mA foot shock, trials interspaced by 2-minute intertrial intervals). On day 2, to examine the conditioned response to the context, the mice were reintroduced to the training context 24 hours later and tested for 5 minutes in the absence of the US. On day 3, in order to examine whether the freezing behavior of the tested mice is associated with a specific context (context A), the mice were reintroduced to a new context (context B) consisting of contextual properties different to context A for 5 minutes. On days 4–7, mice were reintroduced to the context A without US to extinguish association of the specific context A and the US (which is described as a form of new learning).

#### Hind-limb–clasping test

The hind-limb–clasping test, indicative of neurodegeneration, was applied as described before [63]. Briefly, mice were suspended by the middle of the tail and lifted 15 cm above the ground; their behaviors were recorded for 15 seconds. The ambient white light was at 90 Lux. Two separate trials were taken with an intertrial interval of 2 days. Hind-limb clasping was rated from 0 to 3 based on severity: 0 = hind limbs splayed outward and away from the abdomen; 1 = one hind limb retracted inward toward the abdomen for at least 50% of the observation period; 2 = both hind limbs partially retracted inward toward the abdomen for at least 50% of the observation period; 3 = both hind limbs completely retracted inward toward the abdomen for at least 50% of the observation period. Scores of 0.5 were used when appropriate. Hind-limb extension reflex severity scores were calculated by averaging the two separate trials.

### Quantification and statistical analysis

All quantifications throughout the study are based on at least three independent experiments. To quantify the relative immunoblot signals, intensities of protein bands were measured using ImageJ software (version 1.38, National Institutes of Health [NIH]). Signal intensities were then normalized as compared with loading control signal intensities, and the mean of control data points was set to 100%, maintaining the variance.

Fluorescence imaging was carried out as described before [64]. Briefly, for imaging of fluorescent signals, a Laser-Scanning Confocal Microscope Fluoview FV1000 (Olympus Hamburg, Germany) equipped with a 60× objective and Fluoview software version 2.1b was used. For multichannel fluorescent imaging, images were sequentially recorded using identical photomultiplier values throughout all individual experiments. Experiments were replicated at least three times from individual preparations of tissue. Images were analyzed using the MetaMorph software version 7.1 (Universal Imaging, Downingtown, PA, USA). ROIs were defined by the “ROI tool function” throughout multiple frames. Overlay files were separated using the “color separate” function within the software package. Identical ROIs were selected using the “transfer region” function. Brightness was adjusted with the “inclusive thresholding” function to define image thresholds. For measurements of fluorescence intensity, the integrated “morphometry analysis” function was used to assess total and average signal intensities of identical ROIs for each individual channel. Areas of overlapping signals between channels were analyzed with the MetaMorph colocalization tool.

Data were analyzed using Graphpad Prism (Graphpad Software, Inc., San Diego, CA, USA) or SPSS (PASW) Statistics 19.0 (version 19; SPSS, Chicago IL, USA). Statistical significance between groups was determined by one- or two-way ANOVA followed by pairwise comparisons or two-tailed unpaired Student *t* test as indicated in the figure legends. Regarding the behavior data, comparisons between the three genotypes were assessed using one-, two- or three-way ANOVA and included the following between-subject factors: sex, age, bins, and days. Statistical analysis of the different behavior experiments did not show any evidence for interactions or main effects regarding the factor sex. During each experiment, researchers were blind to the genotype, identity of each individual, and treatment. Comparison were considered statistically significant when *p < 0.05, **p < 0.01, and ***p < 0.001. Averaged data are presented as means ± SEM.

Full scanned images of immunoblots are summarized in S1 Raw Images. S1 Protocols provide further details for the generation of the spastin knockout mice; the primary neuronal culture of hippocampal neurons and transfection; genotyping, antibodies, protein extraction, differential centrifugation, and western blot analysis; histology and immunohistochemistry; EM; electrophysiological recordings; and behavioral analysis.

## Supporting information

S1 Fig Related to Fig 1. **Basic characterization and motor analysis of spastin knockout mice.** (A, B) Representative western blot and corresponding quantification of p60 katanin levels in hippocampal lysates from adult mice. NSE used as loading control. *n* = 4 (+/+), *n* = 4 (−/−); Student *t* test. (C) Representative brain sizes. Scale bar, 5 mm. (D) Quantification of the body weight, males, (+/+) *n* = 12, (+/−) *n* = 10, (−/−) *n* = 11. (E) Quantification of the body weight, females, (+/+) *n* = 11, (+/−) *n* = 10, (−/−) *n* = 11. (F) Quantification of the brain/body weight ratio, (+/+) *n* = 3, (+/−) *n* = 5, (−/−) *n* = 5. (G, H) Inverted grid test using 8-month-old (filled bars) or 14-month-old (open bars) mice with body weight as a covariate. Holding impulse (Impulse) = Body weight × Hang time, indicative of muscle grip strength. Eight-month-old mice: main effect for genotype: F_2,58_ = 1.14; *p* = 0.327; followed by pairwise comparison for (+/+) and (+/−) with *p* = 0.344 and (+/+) and (−/−) with *p* = 0.144; 14-month old mice: main effect for genotype: F_2,54_ = 13.49; *p <* 0.0001; followed by pairwise comparison for (+/+) and (+/−) with *p <* 0.001 and (+/+) and (−/−) with *p <* 0.0001. Quantification of the (I) stride length and (J) the gait length during the gait analysis test presented in Fig 1H–1K (14-month-old mice). ANOVA followed by pairwise comparison was used to assess statistical significance. (K, L) DAPI staining of cortical sections of (K) 3-month-old and (L) 13-month-old spastin (+/+) and (−/−) mice presented in Fig 1S–1U; scale bar, 260 μm. ***p < 0.001. Data represented as means ± SEM. Individual quantitative observations that underlie the data presented in this figure are summarized in S8 Data. NSE, neuron specific enolase.
(TIF)

S2 Fig Related to Figs 1 and 2. **Anxiety-related behavior in spastin-depleted mice.** (A, B) Open-field test to study locomotor activity and anxiety-related behavior in spastin mutant mice (+/−, −/−) and their wild-type littermates (+/+). (A) Normal anxiety-like behavior in spastin mutant mice at 8 and 14 months of age revealed by time in the central zone(s). Eight-month-old mice: main effect for genotype: F_2,57_ = 1,263; *p* = 0.291; followed by pairwise comparisons for (+/+) and (+/−) with *p* = 0.279 and (+/+) and (−/−) with *p* = 0.610; 14-month-old mice: main effect for genotype: F_2,44_ = 0.434; *p* = 0.651; followed by pairwise comparisons for (+/+) and (+/−) with *p* = 0.692 and (+/+) and (−/−) with *p* = 0.594. (B) No differences regarding the total distance traveled in the open field (measures locomotor activity) between spastin mutant (+/− and −/−) and wild-type mice (+/+). Eight-month-old mice: main effect for genotype: F_2,57_ = 0.776; *p* = 0.465; followed by pairwise comparisons for (+/+) and (+/−) with *p* = 0.504 and (+/+) and (−/−) with *p* = 0.540; 14-month-old mice: main effect for genotype: F_2,44_ = 0.722; *p* = 0.491; followed by pairwise comparisons for (+/+) and (+/−) with *p* = 0.543 and (+/+) and (−/−) with *p* = 0.552. ANOVA followed by pairwise comparison was used to assess statistical significance. (C, D) Screening of anxiety-like phenotypes by elevated plus maze in 8- and 14-month-old spastin mutant mice (+/− and −/−) and their wild-type littermates (+/+). Normal anxiety levels in all genotypes revealed by the percentage of time spent in open arms. Eight-month-old mice: main effect for genotype: F_2,58_ = 0.305; *p* = 0.738; followed by pairwise comparisons for (+/+) and (+/−) with *p* = 0.620 and (+/+) and (−/−) with *p* = 0.447; 14-month-old mice: main effect for genotype: F_2,45_ = 0.395; *p* = 0.676; followed by pairwise comparisons for (+/+) and (+/−) with *p* = 0.982 and (+/+) and (−/−) with *p* = 0.451. No differences regarding the total distance traveled in the elevated plus maze between spastin mutant (+/− and −/−) and wild type mice (+/+). Eight-month-old mice: main effect for genotype: F_2,58_ = 1.215; *p* = 0.304; followed by pairwise comparisons for (+/+) and (+/−) with *p* = 0.851 and (+/+) and (−/−) with *p* = 0.151; 14-month-old mice: main effect for genotype: F_2,45_ = 1.058; *p* = 0.356; followed by pairwise comparisons for (+/+) and (+/−) with *p* = 0.345 and (+/+) and (−/−) with *p* = 0.161. *p < 0.05, ANOVA followed by pairwise comparison was used to assess statistical significance. (E, F) Light–dark box test showing normal anxiety levels in spastin mutant mice at 8 and 14 months old revealed by percentage of time in the dark box. Eight-month-old mice: main effect for genotype: F_2,58_ = 0.707; *p* = 0.497; followed by pairwise comparisons for (+/+) and (+/−) with *p* = 0.296 and (+/+) and (−/−) with *p* = 0.330; 14-month-old mice: main effect for genotype: F_2,45_ = 0.264; *p* = 0.769; followed by pairwise comparisons for (+/+) and (+/−) with *p* = 0.501 and (+/+) and (−/−) with *p* = 0.577. No differences among the three genotypes regarding number of transitions regardless of the age of the mice. Eight-month-old mice: main effect for genotype: F_2,58_ = 1.863; *p* = 0.164; followed by pairwise comparisons for (+/+) and (+/−) with *p* = 0.214 and (+/+) and (−/−) with *p* = 0.065; 14-month old mice: main effect for genotype: F_2,45_ = 0.034; *p* = 0.967; followed by pairwise comparisons for (+/+) and (+/−) with *p* = 0.916 and (+/+) and (−/−) with *p* = 0.878. 8-month old cohort: (+/+) M = 12 mice, (+/+) F = 11 mice; (+/−) M = 10 mice, (+/−) F = 9 mice; (−/−) M = 10 mice, (−/−) F = 11 mice. Fourteen-month-old cohort: (+/+) M = 8 mice, (+/+) F = 9 mice; (+/−) M = 9 mice, (+/−) F = 7 mice; (−/−) M = 9 mice and (−/−) F = 8 mice. ANOVA followed by pairwise comparison was used to assess statistical significance. Data are represented as means ± SEM. Individual quantitative observations that underlie the data presented in this figure are summarized in S8 Data. F, female; M, male.
(TIF)

S3 Fig Related to Fig 5. **Quantification of fluorescence intensity levels for total tubulin.** (A) Representative coimmunostaining of DAPI (nuclei) and total tubulin in CA1 region using coronal sections. (B) Quantification of the immunofluorescence intensity levels of total tubulin in the CA1 region of the hippocampus. (+/+) *n* = 9 mice; (−/−) *n* = 9 mice. Scale bar, 100 μm. Student *t* test was used to assess statistical significance. Data are represented as means ± SEM. Individual quantitative observations that underlie the data presented in this figure are summarized in S8 Data.
(TIF)

S4 Fig Related to Fig 7. **Spastin depletion impairs AMPAR transport and cell surface delivery.** Quantification of cell surface GluA2 (A) cluster number per 10 μm dendrite length and (J) cluster size in dendrites after spastin depletion presented in Fig 7O and 7P. (+/+), *n* = 19; (−/−), *n* = 19. ***p < 0.001. Student *t* test was used to assess statistical significance. Data are represented as means ± SEM. Individual quantitative observations that underlie the data presented in this figure are summarized in S8 Data. AMPAR, α-amino-3-hydroxy-5-methyl-4-isoxazolepropionic acid receptor; GluA2, glutamate receptor AMPA type subunit 2.
(TIF)

S1 Data Individual quantitative observations that underlie the data presented in Fig 1. The file “S1_data.xlsx” consists of several spreadsheets. Each spreadsheet contains the numerical raw data of one subfigure as indicated.
(XLSX)

S2 Data Individual quantitative observations that underlie the data presented in Fig 2. The file “S2_data.xlsx” consists of several spreadsheets. Each spreadsheet contains the numerical raw data of one subfigure as indicated.
(XLSX)

S3 Data Individual quantitative observations that underlie the data presented in Fig 3. The file “S3_data.xlsx” consists of several spreadsheets. Each spreadsheet contains the numerical raw data of one subfigure as indicated.
(XLSX)

S4 Data Individual quantitative observations that underlie the data presented in Fig 4. The file “S4_data.xlsx” consists of several spreadsheets. Each spreadsheet contains the numerical raw data of one subfigure as indicated.
(XLSX)

S5 Data Individual quantitative observations that underlie the data presented in Fig 5. The file “S5_data.xlsx” consists of several spreadsheets. Each spreadsheet contains the numerical raw data of one subfigure as indicated.
(XLSX)

S6 Data Individual quantitative observations that underlie the data presented in Fig 6. The file “S6_data.xlsx” consists of several spreadsheets. Each spreadsheet contains the numerical raw data of one subfigure as indicated.
(XLSX)

S7 Data Individual quantitative observations that underlie the data presented in Fig 7. The file “S7_data.xlsx” consists of several spreadsheets. Each spreadsheet contains the numerical raw data of one subfigure as indicated.
(XLSX)

S8 Data Individual quantitative observations that underlie the data presented in S1 Fig, S2 Fig, S3 Fig, and S4 Fig. The file “S8_data.xlsx” consists of several spreadsheets. Each spreadsheet contains the numerical raw data of one subfigure as indicated.
(XLSX)

S1 Raw Images Raw images related to Figs 1, 5, 6 and 7 and S1 Fig. Full scanned images of immunoblots. The cropped areas are outlined in broken red lines. References to section figures are indicated. (A, B) Full scans for Fig 1. (C-E) Full scans for Fig 5. (F, G) Full scans for Fig 6. (H-K) Full scans for Fig 7. (L, M) Full scans for S1 Fig. M, marker; x, lanes not included in the final figure.
(TIF)

S1 Protocols The file “S1_protocols.pdf” provides further details for the generation of the spastin knockout mice, the primary neuronal culture of hippocampal neurons and transfection, genotyping, antibodies, protein extraction, differential centrifugation and western blot analysis, histology and immunohistochemistry, electron microscopy, electrophysiological recordings, and behavioral analysis. (PDF)

## Abbreviations

AAA: ATPase associated with various cellular activities
AMPA: α-amino-3-hydroxy-5-methyl-4-isoxazolepropionic acid
AMPAR: AMPA receptor
AMP-PNP: adenylyl-imidodiphosphate
cHSP: complicated HSP
CNS: central nervous system
D-APV: d-2-amino-5-phosphonovaleric acid
DIV: days in vitro
EB3: end-binding protein 3
EGFP: enhanced green fluorescent protein
EM: electron microscopy
EPSC: excitatory postsynaptic current
ER: endoplasmic reticulum
flp: recombinase flippase
frt: flippase recognition target
GFAP: glial fibrillary acidic protein
GFP: green fluorescent protein
GluA2: Glutamate receptor AMPA type subunit 2
HSP: hereditary spastic paraplegia
IQ: intelligence quotient
KIF: kinesin family protein
LacZ: lac operon coding for beta-galactosidase
LFP: local field potential
loxP: locus of X-over P1
MAP: microtubule-associated protein
mEPSC: miniature excitatory postsynaptic current
NBQX: 2,3-dioxo-6-nitro-7-sulfamoyl-benzo[f]quinoxaline
NeoR: neomycin resistance
NMDAR: *N*-methyl-d-aspartate receptor
NSE: neuron specific enolase
pex26: peroxisome assembly protein 26
PGs1: polyglutamylase subunit 1
PSD-95: postsynaptic density protein 95
PTM: posttranslational modification
SA: spontaneous alternation
shRNA: short hairpin RNA
SPG: spastic paraplegia
TBS: theta burst stimulation
+TIP: microtubule plus-end tracking protein
TTLL1: tubulin tyrosin ligase like 1
TUBB3: β3-tubulin
