# Supplementary figures and images for: Spastin depletion increases tubulin polyglutamylation and impairs kinesin-mediated neuronal transport, leading to working and associative memory deficits
Source: PLoS Biol. 2020 Aug 31;18(8):e3000820. doi: 10.1371/journal.pbio.3000820 (PMC7485986; doi:10.1371/journal.pbio.3000820)

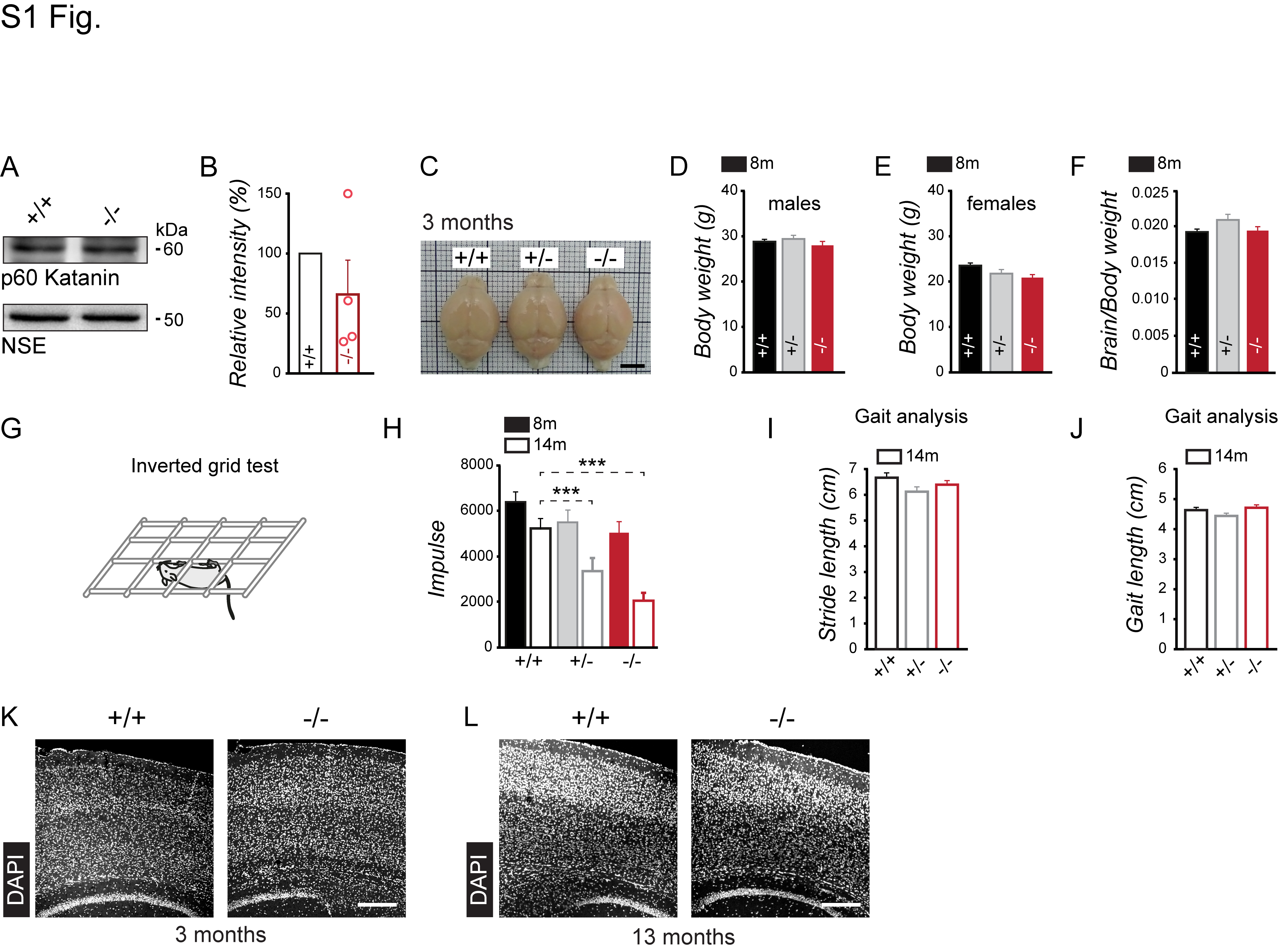

Supplement: S1 Fig — Basic characterization and motor analysis of spastin knockout mice. (A, B) Representative western blot and corresponding quantification of p60 katanin levels in hippocampal lysates from adult mice. NSE used as loading control. n = 4 (+/+), n = 4 (−/−); Student t test. (C) Representative brain sizes. Scale bar, 5 mm. (D) Quantification of the body weight, males, (+/+) n = 12, (+/−) n = 10, (−/−) n = 11. (E) Quantification of the body weight, females, (+/+) n = 11, (+/−) n = 10, (−/−) n = 11. (F) Quantification of the brain/body weight ratio, (+/+) n = 3, (+/−) n = 5, (−/−) n = 5. (G, H) Inverted grid test using 8-month-old (filled bars) or 14-month-old (open bars) mice with body weight as a covariate. Holding impulse (Impulse) = Body weight × Hang time, indicative of muscle grip strength. Eight-month-old mice: main effect for genotype: F2,58 = 1.14; p = 0.327; followed by pairwise comparison for (+/+) and (+/−) with p = 0.344 and (+/+) and (−/−) with p = 0.144; 14-month old mice: main effect for genotype: F2,54 = 13.49; p < 0.0001; followed by pairwise comparison for (+/+) and (+/−) with p < 0.001 and (+/+) and (−/−) with p < 0.0001. Quantification of the (I) stride length and (J) the gait length during the gait analysis test presented in Fig 1H–1K (14-month-old mice). ANOVA followed by pairwise comparison was used to assess statistical significance. (K, L) DAPI staining of cortical sections of (K) 3-month-old and (L) 13-month-old spastin (+/+) and (−/−) mice presented in Fig 1S–1U; scale bar, 260 μm. ***p < 0.001. Data represented as means ± SEM. Individual quantitative observations that underlie the data presented in this figure are summarized in S8 Data. NSE, neuron specific enolase. (TIF) [file pbio.3000820.s001.tif]

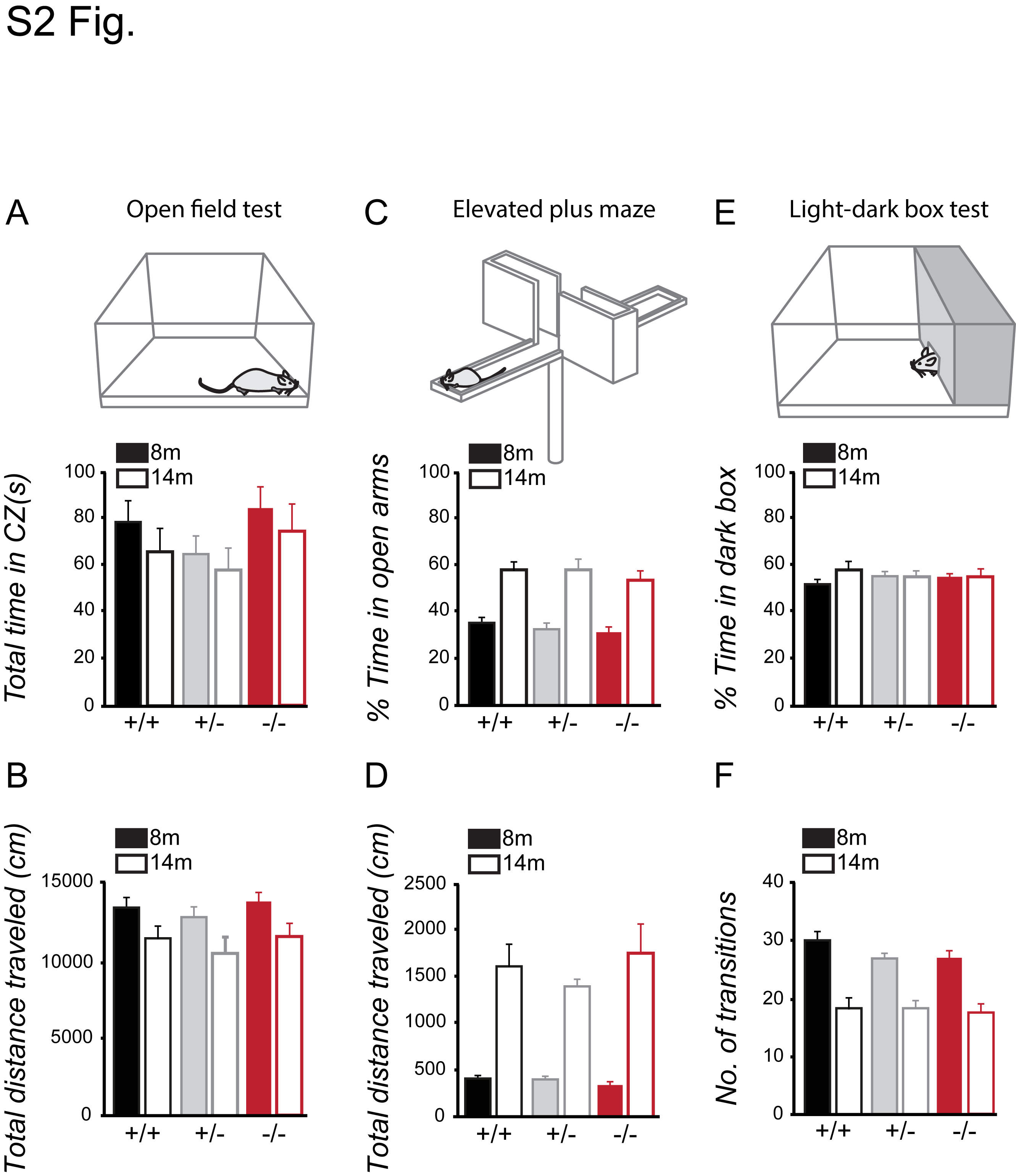

Supplement: S2 Fig — Anxiety-related behavior in spastin-depleted mice. (A, B) Open-field test to study locomotor activity and anxiety-related behavior in spastin mutant mice (+/−, −/−) and their wild-type littermates (+/+). (A) Normal anxiety-like behavior in spastin mutant mice at 8 and 14 months of age revealed by time in the central zone(s). Eight-month-old mice: main effect for genotype: F2,57 = 1,263; p = 0.291; followed by pairwise comparisons for (+/+) and (+/−) with p = 0.279 and (+/+) and (−/−) with p = 0.610; 14-month-old mice: main effect for genotype: F2,44 = 0.434; p = 0.651; followed by pairwise comparisons for (+/+) and (+/−) with p = 0.692 and (+/+) and (−/−) with p = 0.594. (B) No differences regarding the total distance traveled in the open field (measures locomotor activity) between spastin mutant (+/− and −/−) and wild-type mice (+/+). Eight-month-old mice: main effect for genotype: F2,57 = 0.776; p = 0.465; followed by pairwise comparisons for (+/+) and (+/−) with p = 0.504 and (+/+) and (−/−) with p = 0.540; 14-month-old mice: main effect for genotype: F2,44 = 0.722; p = 0.491; followed by pairwise comparisons for (+/+) and (+/−) with p = 0.543 and (+/+) and (−/−) with p = 0.552. ANOVA followed by pairwise comparison was used to assess statistical significance. (C, D) Screening of anxiety-like phenotypes by elevated plus maze in 8- and 14-month-old spastin mutant mice (+/− and −/−) and their wild-type littermates (+/+). Normal anxiety levels in all genotypes revealed by the percentage of time spent in open arms. Eight-month-old mice: main effect for genotype: F2,58 = 0.305; p = 0.738; followed by pairwise comparisons for (+/+) and (+/−) with p = 0.620 and (+/+) and (−/−) with p = 0.447; 14-month-old mice: main effect for genotype: F2,45 = 0.395; p = 0.676; followed by pairwise comparisons for (+/+) and (+/−) with p = 0.982 and (+/+) and (−/−) with p = 0.451. No differences regarding the total distance traveled in the elevated plus maze between spastin mutant (+/− [file pbio.3000820.s002.tif]

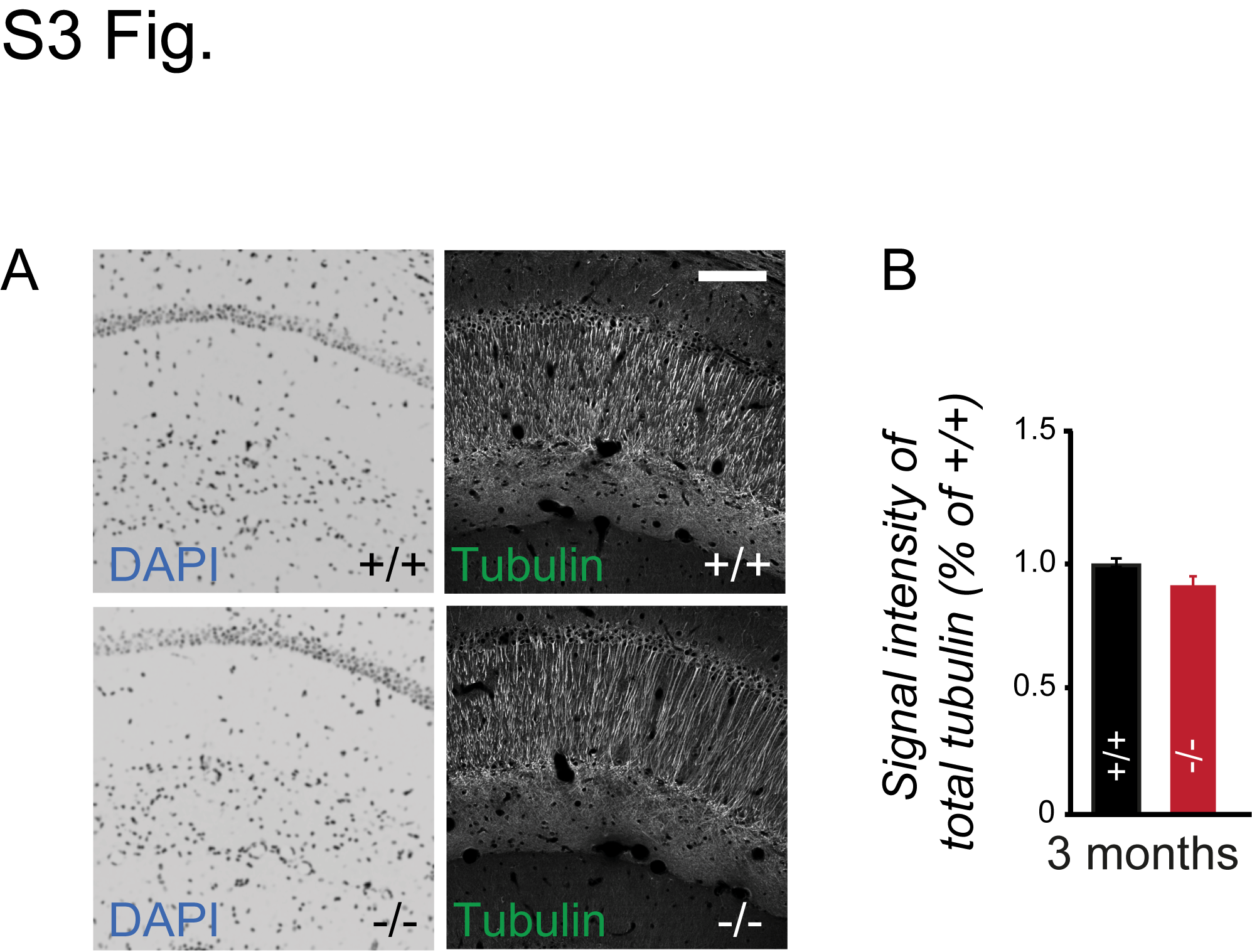

Supplement: S3 Fig — Quantification of fluorescence intensity levels for total tubulin. (A) Representative coimmunostaining of DAPI (nuclei) and total tubulin in CA1 region using coronal sections. (B) Quantification of the immunofluorescence intensity levels of total tubulin in the CA1 region of the hippocampus. (+/+) n = 9 mice; (−/−) n = 9 mice. Scale bar, 100 μm. Student t test was used to assess statistical significance. Data are represented as means ± SEM. Individual quantitative observations that underlie the data presented in this figure are summarized in S8 Data. (TIF) [file pbio.3000820.s003.tif]

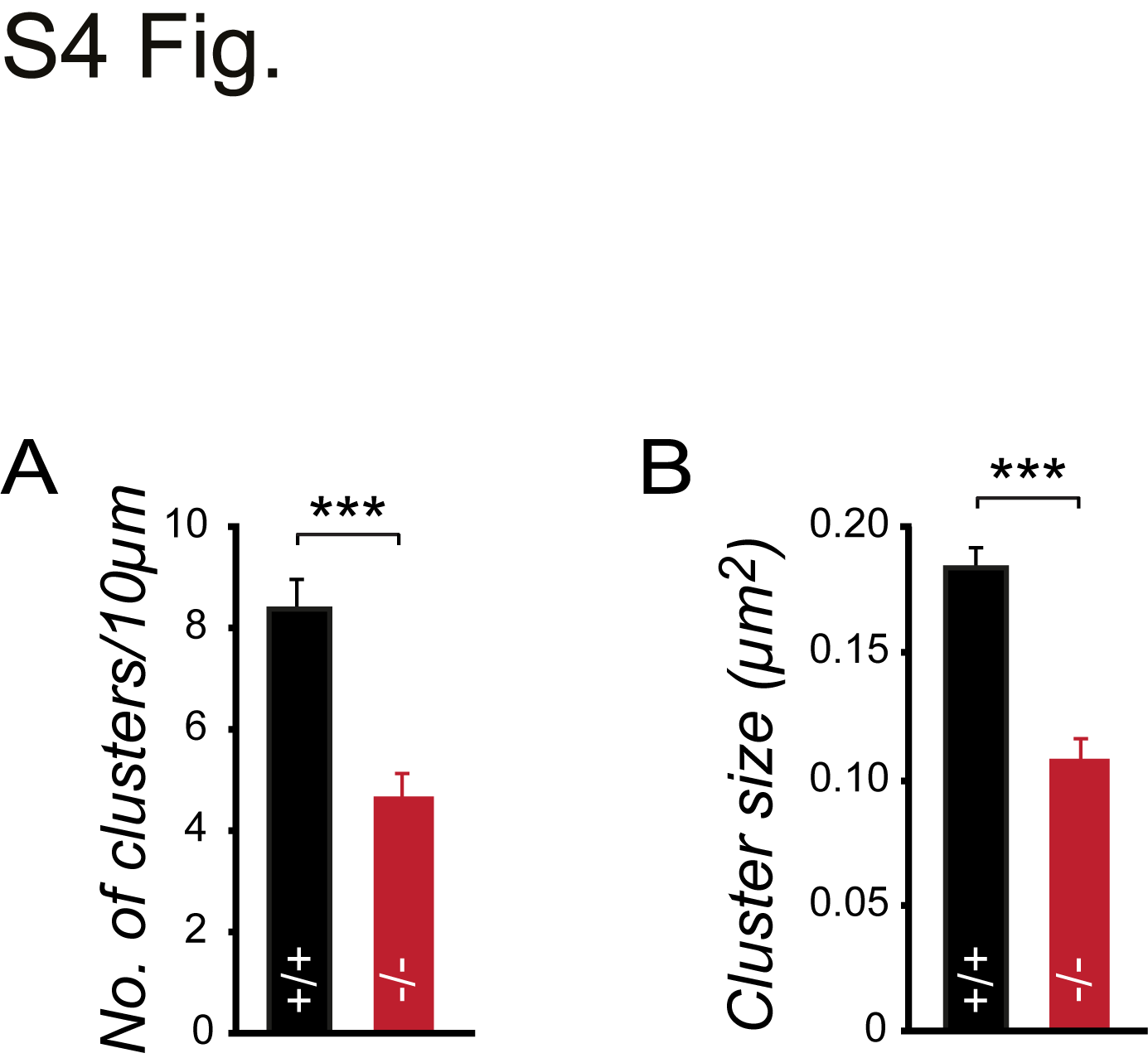

Supplement: S4 Fig — Spastin depletion impairs AMPAR transport and cell surface delivery. Quantification of cell surface GluA2 (A) cluster number per 10 μm dendrite length and (J) cluster size in dendrites after spastin depletion presented in Fig 7O and 7P. (+/+), n = 19; (−/−), n = 19. ***p < 0.001. Student t test was used to assess statistical significance. Data are represented as means ± SEM. Individual quantitative observations that underlie the data presented in this figure are summarized in S8 Data. AMPAR, α-amino-3-hydroxy-5-methyl-4-isoxazolepropionic acid receptor; GluA2, glutamate receptor AMPA type subunit 2. (TIF) [file pbio.3000820.s004.tif]

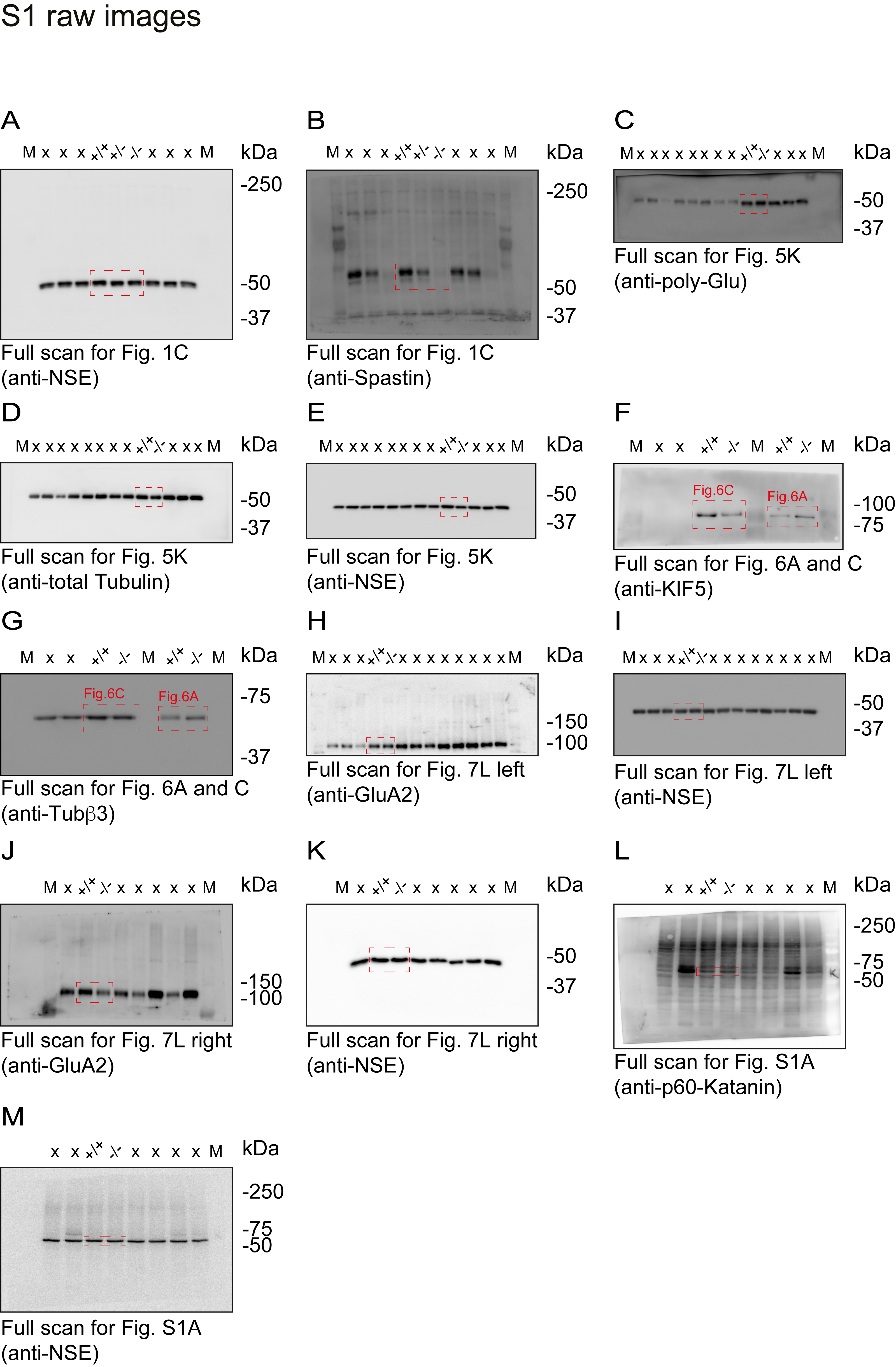

Supplement: S1 Raw Images — Full scanned images of immunoblots. The cropped areas are outlined in broken red lines. References to section figures are indicated. (A, B) Full scans for Fig 1. (C-E) Full scans for Fig 5. (F, G) Full scans for Fig 6. (H-K) Full scans for Fig 7. (L, M) Full scans for S1 Fig. M, marker; x, lanes not included in the final figure. (TIF) [file pbio.3000820.s013.tif]
