# Supplementary material for: Spastin depletion increases tubulin polyglutamylation and impairs kinesin-mediated neuronal transport, leading to working and associative memory deficits
Source: PLoS Biol. 2020 Aug 31;18(8):e3000820. doi: 10.1371/journal.pbio.3000820 (PMC7485986; doi:10.1371/journal.pbio.3000820)
Supplement: S1 Protocols — (PDF) [file pbio.3000820.s014.pdf]

Supporting Protocols

Generation of Spastin Knockout Mice. Generation of spastin knockout mice ($Spast^{-/-}$) was previously described [1]. Briefly, Spastin +/+, +/- and -/- mice derived from heterozygous matings were bred in a specific-pathogen-free (SPF) animal facility. Mice (male and female; both sexes used for all experiments) were group-housed (2-5 mice per cage) under a reversed 12-hour light/dark cycle and divided into 2 different experimental cohorts: 8- and 14-months old at the beginning of the behavioral assessment. Temperature (22±1 °C) and humidity (50±5%) in the animal facility were kept constant, and the animals had *ad libitum* access to food and water. The behavioral experiments were conducted during the dark phase of the light cycle. The composition of the middle-aged cohort (8 months) was the following: 23 +/+ mice; 19 +/- mice and 22 -/- mice. The aged cohort of mice (14 months) consisted of 23 +/+ mice, 21 +/- and 15 -/- mice. The behavioral testing sequence was the following: elevated plus maze (1 day), open field test (1 day), light-dark box (1 day), inverted grid test (1 day), pole test (14 month old mice only, 1 day), accelerating rotarod (3 days), beam walking test (14 month old mice only, 4 days), Y-maze spontaneous alternation (2 days), T-maze confined alternation (2 days) and contextual fear conditioning (7 days). The composition of a second aged cohort of mice (14 months) consisted of 15 +/+ mice, 17 +/- and 14 -/- mice. The behavioral testing sequence was the following: Gait analysis test (1 day) and hind limb clasping test (4 days).

Primary Neuronal Culture of Hippocampal Neurons and Transfection. 12 mm glass coverslips (Carl Roth, Karlsruhe, Germany) were coated with Poly-L-lysine (Sigma-Aldrich, Buchs, Switzerland) (50 µg/ml). Hippocampi of mice embryonic day E16 were isolated and trypsinized in 0.05% trypsin/EDTA for 5 minutes at 37°C (Invitrogen, Waltham, MA, USA). Hippocampi were dissociated in HEPES buffer (10 mM HEPES, 135 mM NaCl, 5 mM KCl, 2 mM $CaCl_2$, 2 mM $MgCl_2$, 15 mM Glucose; pH 7.4). 60,000 cells were plated per well and incubated at 37 °C and 5% CO_2. For transfection of DIV 5-11 hippocampal neurons 2 µg plasmid DNA was diluted in 25 µl of sterile H_2O, containing 6.5 µl 1 M $CaCl_2$ solution. This DNA solution was added dropwise to 25 µl 2x HBS under vortex agitation and incubated at

room temperature (RT) for 10 minutes. Neuronal cells were incubated with the DNA solution for 1h at 37ºC with 5% CO_2. The calcium precipitate was removed by rinsing the cells twice with pre-warmed HEPES buffer. Hippocampal neurons were cultured in the original (conditioned) medium for indicated time points at 37ºC and 5% CO_2.

Genotyping. Genomic DNA was isolated from tail biopsies using the Quick Extract Buffer (Biozym Scientific GmbH, Hessisch Oldendorf, Germany). For genotyping of spastin +/+, +/- and -/- 3 spastin oligonucleotides were used (PLR41: AAGTCATGGCAGTCTTTCTGGCT; PLR89: CACATGGTGGCTCATAACCATTTA; PLR169: ATTTGCAAAAACTACTTGCTATTAAATCC). PCR product sizes: 223 base pairs for the wild type allele and 432 base pairs for the spastin knockout allele.

Antibody Application Details. The following antibodies were used for immunofluorescence or western blotting analysis: mouse anti-α-tubulin (Abcam, Cambridge, UK; AB7291, WB 1/5000), mouse anti-polyglutamylated tubulin (GT335) (Adipogen, Liestal, Switzerland; AG-20B-0020, ICC 1/500, IHC 1/200, WB 1/5000), rabbit anti-tubulin α4A (Abcam, Cambridge, UK; AB177479, IHC 1/200), mouse anti-tubulin-βIII (Biolegend, San Diego, CA, USA; 801201, WB 1/10 000), mouse anti-PSD95 (Thermo Fisher Scientific, MA, USA; MA1-046, ICC 1/2000), guinea pig anti-synaptophysin-1 (Synaptic Systems, Göttingen, Germany; 101004, ICC 1/2000, IHC 1/600), guinea pig anti-GFAP (Synaptic Systems, Göttingen, Germany; 173-004, IHC 1:300), mouse anti-GluA2 (Millipore, Darmstadt, Germany; MAB397, ICC 1/300, WB 1/500), rabbit anti-KIF5c (Thermo Fischer Scientific, MA, USA; PA1-644, WB 1/500), mouse anti-KIF1a (BD Bioscience Laboratories, San Jose, CA, USA; 612094, WB 1/1000), mouse anti-human katanin p60 (R&D Systems, Minneapolis, MN, USA; MAB7100, WB 1/500), mouse anti-spastin (Abcam, Cambridge, UK; AB77144, WB 1/500), chicken anti-neuronal-specific enolase (Novus Biologicals, Littleton, CO, USA; NB100-046, WB 1/5000), mouse HRP-conjugated (Dianova, Hamburg, Germany; 715-036-151, WB 1/10 000), rabbit HRP-conjugated (Dianova, Hamburg, Germany; 711-036-152, WB 1/10 000), chicken HRP-

conjugated (Dianova, Hamburg, Germany; 103-035-155, WB 1/2500), mouse Cy3 (Dianova, Hamburg, Germany; 715-165-150, ICC 1/500), rabbit AF488 (Dianova, Hamburg, Germany; 711-545-152, ICC 1/500), guinea pig Cy5 (Dianova, Hamburg, Germany; 706-176-148, ICC 1/500). Nuclei were visualized with DAPI (Merck, Darmstadt, Germany; 124653, ICC 1/1000) and F-actin with rhodamine-conjugated Phalloidin (Cytoskeleton, Denver, CO, USA; PHDR1, ICC 1/500).

Protein Extraction, Differential Centrifugation and Western Blot Analysis. Spastin +/+ and -/- mice were sacrificed using CO_2 and brains were isolated and placed in 1.5 ml ice-cold homogenization buffer (20 mM HEPES, 100 mM potassium acetate, 40 mM KCl, 5 mM EGTA, 5 mM $MgCl_2$, pH 7.2, protease inhibitor (Roche Complete, Sigma-Aldrich, Hamburg, Germany), 2 mM mg ATP, 5 mM DTT and 1 mM PMSF) and homogenized using a tissue grinder. Samples were centrifuged for 10 minutes at 1000 x *g* (JA 20 or JS 13.1 Beckman, Brea, CA, USA). The supernatant (S1 termed: total protein fraction) was centrifuged for 10 minutes at 10 000 x *g* (JA 20 or JS 13.1 Beckman) and pellet (P1) was discarded. The supernatant was collected into a new tube (S2) and the pellet (P2 termed: plasma membran-enriched fraction) was resuspended in homogenization buffer. The collected samples were diluted with 4 x sample buffer and boiled at 95 °C for 10 minutes, and either used immediately or stored at -80 °C. Equal amounts of protein from hippocampal lysates (10 µg) were subjected to SDS-PAGE and transferred to polyvinylidene difluoride (PVDF). Primary Antibody was added for 60 minutes at room temperature or overnight at 4 °C and after washing incubated for 60 minutes with the horseradish peroxidase-conjugated (HRP) secondary antibody. Bands were visualized by a chemiluminescence detection system (INTAS Chemo Cam 3.2, Göttingen, Germany). The acquired images were processed using Adobe Photoshop 6.0.1 and the intensities of individual bands were measured using the ImageJ software (ImageJ 1.50i, NIH, USA).

Histology and Immunohistochemistry. Animals were anesthetized and perfused transcardially with ice-cold Saline (0.9% NaCl) followed by 4% phosphate-buffered paraformaldehyde solution (PFA). Brains were removed and post-fixed for 4 hours in the same fixative at 4 °C, cryopreserved over two days in 30% sucrose, and frozen on dry ice. The frozen brains were mounted using Tissue Tek (VWR, PA, USA) and sectioned into 30 µm coronal or 20µm sagital sections using a cryostat (Leica CM3050, Wetzlar, Germany).

Nissl staining.: The brain sections were mounted on glass slides and stored at -20 °C. Coronal sections were washed three times for 15 minutes in 1x PBS and stained using 0.5% cresyl violet staining solution containing a few drops of glacial acetic acid for 15 minutes at room temperature. After the incubation, sections were rinsed in H_2O and dehydrated successively with 70%, 95% and 100% ethanol. Following dehydration, the brain slices were immersed in xylene and mounted using ENTELLAN® mounting medium (Merck, Darmstadt, Germany). Sections were analyzed using the ImageJ software (NIH, Bethesda, MD, USA).

Immunohistochemistry of tissue sections: sagital sections were collected in PBS with 0.01% NaAc. For staining sections were incubated in PBS + 1% Triton X-100 for 30 minutes at RT and then overnight at 4 °C in primary antibodies dissolved in PBS. Secondary antibodies were added for 3 h at room temperature (RT). They were rinsed further in PBS and finally mounted in Aqua Poly Mount (Polysciences, Eppelheim, Germany).

Immunohistochemistry to detect poly-Glu tubulin

Frozen tissue from +/+ and -/- mice were embedded in 4% agarose and sectioned into 30 µm coronal sections using a Vibratome (Leica VT 1000S, Wetzlar, Germany). Brain slices were collected in PBS and stored at -20 °C in antifreeze solution (50 mM phosphate buffer, 37.5% (v/v) ethylene glycol and sucrose) followed by fluorescent immunohistochemical staining. Brain sections were rinsed three times in PBS and permeabilized in 1% Triton-X-100 (v/v) in PBS for 10 minutes. Antigen blocking was carried out by incubating the sections with 1% Bovine Serum Albumin (BSA) in PBS for one hour, followed by rinsing with PBS and incubation

overnight at 4 °C in a humid chamber in primary antibodies diluted in the blocking buffer. On the following day, the sections were rinsed three times with PBS prior to incubation in secondary antibodies diluted in the blocking buffer for 2 hours at room temperature (RT). Afterwards, the sections were rinsed further in PBS and finally mounted in Aqua-Poly/Mount coverslipping medium (Polysciences, Eppelheim, Germany).

Immunocytochemistry. Cultured hippocampal neurons were fixed in 4% paraformaldehyde/ 4% sucrose diluted in PBS for 10 minutes. Cells were permeabilized for 4 minutes in PBS containing 0.5% triton-X-100, and blocked in PBS containing 1% (w/v) BSA. Neurons were incubated for one hour in primary antibodies diluted in blocking buffer at RT or overnight at 4 °C. Secondary antibodies and DAPI were added for one hour. Coverslips were mounted in mounting media. This protocol was followed for all the standard immunocytochemistry, except for surface GluA2 staining, when cells were incubated with the primary antibody under non-permeabilizing conditions for 2 hours at room temperature followed by permeabilization for 4 minutes and blocking with 1% (w/v) BSA in PBS.

Electron Microscopy. Adult spastin +/+ and -/- mice were anesthetized and transcardially perfused with a mixture of 4% paraformaldehyde and 1% glutaraldehyde in 0.1 M phosphate buffer at pH 7.4. The brains were processed using a vibratome (Leica VT 1000S, Wetzlar, Germany) to obtain 100 µm thick sections. These sections were rinsed three times in 0.1 M sodium cacodylate buffer (pH 7.2-7.4) (Sigma-Aldrich, Buchs, Switzerland) and incubated with 1% osmium tetroxide (Science Services, Munich, Germany) in cacodylate buffer for 20 minutes on ice. The osmication of sections was followed by dehydration through ascending ethyl alcohol concentration steps and rinsed twice in propylene oxide (Sigma-Aldrich, Buchs, Switzerland). Infiltration of the embedding medium was performed by immersing the pieces first in a mixture of 2:1 of propylene oxide and Epon (Carl Roth, Karlsruhe, Germany) then in a 1:1 mixture and finally in neat Epon and hardened at 60 °C for 48 hours. Ultrathin sections (60 nm) were observed using an EM902 transmission electron microscope (Zeiss, Germany)

equipped with a CCD in lens 2K digital camera and running the ImageSP software (Tröndle, Moorenweis, Germany). Primary hippocampal neurons, cultured on Aclar (Ted Pella, Redding, USA), were fixed, embedded and polymerized on already polymerized Epon slides. Thereafter the Aclar (Ted Pella, Redding, USA) was drawn off and ultrathin sections were prepared and investigated as described above. Defined microtubules in dendrites were traced using the ImageJ software. For ultrastructural analysis in the CA1 region of the hippocampus, only synapses with intact synaptic plasma membranes with recognizable pre- and postsynaptic density and defined synaptic vesicle membranes were used. Synaptic vesicles were classified as docked if they touched the synaptic vesicle membrane and the membrane of the active zone. For each sample, the number of docked, undocked and the total number of synaptic vesicles per synapse as well as the total number of synapses were analyzed within a square of 18 μm^2, randomly placed in the CA1 region of the hippocampus.

Electrophysiological Recordings. *Recordings in brain slices.* Animals were anaesthetized with isofluorane and decapitated. Hippocampal coronal sections were prepared using a vibroslicer (VT1200S, Leica, Bensheim, Germany). Throughout the experiments, brain slices were superfused with artificial cerebrospinal fluid (ACSF) containing (in mM): NaCl, 120; $NaHCO_3$, 26; NaH_2PO_4, 1; KCl, 2,5; D-glucose, 2,8; $CaCl_2$, 4; $MgCl_2$, 1. ACSF was continuously gased with 5%/95% CO_2/O_2 to adjust the pH to 7.4 to maintain viability of the cells. Drugs were applied with the perfusion system. CA3 pyramidal cells were investigated using the patch-clamp technique (EPC 9 amplifier and Patch master 2.65 software, HEKA, Lambrecht, Germany). The whole-cell configuration was established using patch pipettes with a resistance of ~3 MΩ, filled with a pipette solution containing (in mM): 4-AP, 5; CsCl, 120; EGTA, 0.2; HEPES, 10; TEA-Cl, 20; $MgCl_2$, 2; $CaCl_2$, 0.5; Na-ATP, 2; Na-GTP, 0.5, pH 7.2. Recordings were digitized (EPC 9, HEKA) at 10-20 kHz and filtered (Bessel filter, 2 kHz). To elicit synaptic currents in CA3 pyramidal cells, mossy fibers were electrically stimulated using a stimulation pipette and an isolated stimulator (DS3, Digitimer, Welwyn Garden City, UK). The stimulation pulse (0.1 ms, 20-50 µA) was increased in amplitude until complex, multiphasic current

responses were induced and was then adjusted closely below the threshold of complex synaptic currents to enable recordings of monophasic synaptic currents with maximal amplitudes. Long-term potentiation (LTP) was investigated in the CA1 region of acute hippocampal brain slices. Field potentials were recorded using a glass electrode (2.5 MΩ) in the dendritic layer of CA1 pyramidal cells. Schaffer collaterals were stimulated using an isolated stimulator (DS3, Digitimer, Welwyn Garden City, UK). fEPSP were digitized (EPC9, HEKA) at 20 kHz and filtered (Bessel filter, 3 kHz). The rising slope of the fEPSP was analyzed to assess changes in synaptic efficacy.

Behavioral Analysis.

Inverted grid test. The apparatus consists of a horizontal square metallic grid with the edges covered. The grid was mounted 40 cm above the floor, supported by a metallic vertical bar in a brightly illuminated room (70 lux). A cage filled with saw dust was placed on the floor as a cushion for the falling mouse. Once the mouse firmly grabbed on the grid with all four paws, the apparatus was carefully inverted. Each experiment was stopped after 300 seconds. The mouse was video-controlled using a fixed video camera (Sony HDR-CX240E, Tokyo, Japan). Performance was evaluated by measuring the latency to fall.

Accelerating Rotarod Test. Mice walked on a rotating rod (Acceler, Rotarod for mice, Jones & Roberts, TSE Systems, Bad Homburg, Germany) at constant speed (4 rpm) for three minutes. After two acclimatization trials, each mouse was placed on the rotating rod for four test trials, during which the rotation speed gradually increased from 4 to 40 rpm within four minutes. The inter trial interval (ITI) was 1h. Performance was evaluated by measuring the latency to fall.

Pole test. Mice were placed on top of a 50 cm vertical, 0.9 cm diameter rod while grasping it with the four paws and the head pointing upwards. 24 hours before the testing day, mice were habituated to the pole by performing three consecutive trials with an inter-trial interval of 30 seconds. In order to motivate the mice to climb down, nesting material from the home cage

was placed at the bottom of the rod. On the test day, each mouse performed 3 trials of max. 90 seconds with a minimal ITI and the best performance was used for analysis. Trials were excluded when the mouse slid down or jumped from the top of the pole. The time each mouse took to make a turn of 180° with the head pointing downwards (time to turn) was quantified for each trial.

Gait analysis test. To assess gait and locomotion, mice were placed in a corridor (53.5 x 5.3 cm) within a box of 55 cm length x 16 cm width x 15 cm height made from acrylic glass with a wall thickness of 5mm. The box was placed 70 cm above ground and equipped with a LED stipe (12 V RGB, 60 LED/ meter) facing inwards around the bottom edge. The ambient red light was at 5 Lux. Two cameras were used to track the mice via Ethovision (version XT 8.5, Noldus Technology, Wageningen, The Netherlands) from the top and record the green illuminated footsteps from the bottom. The mice were allowed to explore for 100 sec. The total distance moved and their latency was calculated from Ethovision files while the gait and stride lengths were measured using Tracker 5.0.7 (Open Source Physics, NSF, Virgina).

Beam walking test. The apparatus consists of 60 cm long rod with a squared or round shape of 9, 11 and 15 mm (edge length or diameter, respectively) with 50 cm off the ground sustained on two poles and illuminated by a light bulb (200 lux). An enclosed box is positioned at the end of the beam as finish point. Each mouse was trained for 3 consecutive days (3 trials per day, with an ITI of 1 minute) to cross the horizontal beam and enter into the enclosed box. On the testing day, each mouse was tested in two consecutive trials from the widest to the narrowest beam, from the squared beam to the round shape beam. Motor performance was recorded using a video camera (Sony HDR-CX240E, Tokyo, Japan). The time to cross the narrowest round beam over two trials as well as the number of foot slips was manually analyzed based on video recordings.

Y-maze spontaneous alternation. The apparatus consists of a Y-shaped maze with three arms arranged in 120° position with identical plastic opaque walls 39 x 9 x 16 cm. The apparatus was placed in a room with dim lightning (10 lux). An arm entry was recorded manually when the mouse moved beyond the central triangle and entered the arm with all four paws. Each mouse was randomly placed into one arm and allowed to move freely through the maze over 10 minutes. Alternation behavior was defined as consecutive entries into each of the three arms in overlapping triplet sets (e.g.: 1, 2, 3 or 2, 1, 3 or 3, 2, 1). The percentage of alternation was calculated as the percentage of actual alternations to the maximum possible number of arm entries.

T-maze confined alternation. The apparatus was made of three opaque plastic arms (30 x 6 cm). The start arm, enclosed by walls 16 cm high, was perpendicular to two opposing arms. The whole apparatus was elevated 40 cm above the floor. On the training trial, the mouse was removed from the home cage and placed onto the distal end of the enclosed arm facing the interception of the arms and was allowed to explore the enclosed arm. As soon as the mouse entered one of the arms by placing all four paws into the arm, the operator isolated the mouse in the chosen arm for 30 seconds using a removable wall, perpendicular to the arm walls. After 30 seconds the mouse was put back into the home cage and subsequently placed back into the starting arm in order to choose between left and right arm. The number of arm entries was recorded in order to calculate the alternation score (number of alternations divided by the maximum number of all possible alternations).

Elevated Plus Maze. The apparatus consists of four equally spaced arms (30 x 16 x 5 cm) made of waterproof polyvinyl (PVC) material and a white plastic floor elevated 80 cm above the ground assembled in a room with diffuse dim lightning (70 lux). Two opposing arms were enclosed with opaque walls whereas the open arms were limited by an edge border of 2 mm height, radiating from a central zone 5 x 5 cm. Anxiety behavior was recorded by a video camera coupled to a computer running the Ethovision XT8.5 tracking system (version XT 8.5,

Noldus Technology, Wageningen, The Netherlands). Briefly, each mouse was removed from its home cage and gently placed in the central zone of the maze facing one of the open arms. Each tested mouse was allowed to freely explore the four arms and the behavior was recorded for 5 minutes. After the test, mice were removed and placed back to the home cage. The analyzed parameters were the following: total distance traveled over 5 minutes and percentage of time exploring the open arms.

Open field test. The apparatus consists of four identical arenas (50 x 50 x 40 cm) made of PVC foam material with a white floor and individually illuminated by a lightning bulb (70 lux). The center (16 x 16 cm) was pre-defined as the central zone. The locomotor activity was monitored online by a video camera coupled to a computer running the Ethovision tracking system (version XT 8.5, Noldus Technology, Wageningen, The Netherlands). Tested mice were placed in the center of the open field and allowed to freely explore the arena for 30 minutes. At the end of each trial, the mouse was returned to the home cage and the arena was cleaned. Total distance traveled in 30 minutes and percentage of time spent in the central zone were used as measures for locomotor activity and anxiety levels, respectively.

Light-dark box. The apparatus consists of an open chamber (45 x 20 x 20 cm) brightly illuminated (340 lux) comprising two thirds of the total testing area and an enclosed chamber (20 x 20 x 15 cm) with a small entry (8 x 7 cm) allowing the mouse to walk freely between two chambers. On the test day, mice were placed onto one of the back corners of the light chamber and video-taped for the next 10 minutes (Sony HDR-CX240E, Tokyo, Japan). The latency to enter the dark side and the total time spent in the dark side were analyzed.

Supporting References

1. Brill MS, Kleele T, Ruschkies L, Wang M, Marahori NA, Reuter MS, et al. Branch-Specific Microtubule Destabilization Mediates Axon Branch Loss during Neuromuscular Synapse Elimination. Neuron. 2016;92(4):845-56. doi: 10.1016/j.neuron.2016.09.049. PubMed PMID: 27773584; PubMed Central PMCID: PMCPMC5133389.
